# Comprehensive Analysis of Immune Implication and Prognostic Value of *IFI44L* in Non-Small Cell Lung Cancer

**DOI:** 10.3389/fonc.2021.798425

**Published:** 2022-01-03

**Authors:** Yong Zeng, Zhe Zhang, Hongqiang Chen, Jun Fan, Wenbo Yuan, Jingzhi Li, Shimeng Zhou, Wenbin Liu

**Affiliations:** ^1^ Institute of Toxicology, College of Preventive Medicine, Army Medical University (Third Military Medical University), Chongqing, China; ^2^ Department of Environmental Health, College of Preventive Medicine, Army Medical University (Third Military Medical University), Chongqing, China; ^3^ Department of Breast and Thyroid Surgery, Daping Hospital, Army Medical University (Third Military Medical University), Chongqing, China; ^4^ School of Public Health, Xinxiang Medical University, Xinxiang, China; ^5^ School of Public Health, China Medical University, Shenyang, China

**Keywords:** *IFI44L*, non-small cell lung cancer, tumor-infiltrating immune cells, prognosis, nomogram

## Abstract

Interferon-induced protein 44-like (*IFI44L*), a type I interferon-stimulated gene (ISG), has been reported to be involved in innate immune processes and to act as a tumor suppressor in several cancers. However, its immune implication on lung cancer remains unclear. Here, we systemically analyzed the immune association of *IFI44L* with multiple tumor-infiltrating immune cells (TIICs) and immunomodulators through bioinformatics methods in The Cancer Genome Atlas (TCGA) lung cancer cohorts. Then, the *IFI44L*-related immunomodulators were selected to construct the prognostic signatures in the lung adenocarcinoma (LUAD) cohort and the lung squamous cell carcinoma (LUSC) cohort, respectively. Concordance index and time-dependent receiver operating characteristics (ROC) curves were applied to evaluate the prognostic signatures. GSE72094 and GSE50081 were used to validate the TCGA-LUAD signature and TCGA-LUSC signature, respectively. A nomogram was established by risk score and clinical features in the LUAD cohort. Finally, the prognostic value and biological function of *IFI44L* were verified in a real-world cohort and *in vitro* experiments. The results indicated that *IFI44L* showed significant correlation with TIICs in LUAD and LUSC samples. Functional enrichment analysis showed that *IFI44L* may participate in various cancer/immune-related pathways, including JAK/STAT signaling pathway and NF-κB signaling pathway. A total of 44 immunomodulators presented obvious association with *IFI44L* in the TCGA-LUAD cohort and a robust 10-immunomodulator signature was constructed. Patients in the higher-risk group presented worse prognosis than those in the lower-risk group. Notably, the risk signature was successfully validated in GSE72094. Multivariate Cox regression suggested that the risk signature could act as independent prognostic factors in both TCGA-LUAD and GSE72094 cohorts. Besides, a 17-immunomodulator signature was established in the TCGA-LUSC cohort and similar results were presented through analysis. The nomogram exhibited good accuracy in predicting overall survival (OS) outcome among TCGA-LUAD patients than the risk signature and other clinical features, with the area under curve values being 0.782 at 1 year, 0.825 at 3 years, and 0.792 at 5 years. Finally, tissue microarray analysis indicated that higher expression of *IFI44L* presented opposite relationship with pathological stage (*p* = 0.016) and a better outcome among lung cancer patients (*p* = 0.024). Functional experiments found that *IFI44L* overexpression significantly inhibited the proliferation, migration, and invasion in LUAD and LUSC cells; RT-qPCR experiments verified the correlation between the expression level of *IFI44L* with multiple immunomodulators in SPC-A-1 and NCI-H520 cells. In conclusion, our research highlighted that *IFI44L* is associated with tumor immune infiltration and provided information on *IFI44L*’s immune implication, which indicates that *IFI44L* has potential clinical immunotherapeutic value and the proposed nomogram is a promising biomarker for non-small cell lung cancer patients.

## Introduction

Lung cancer (LC) is the most common cause of cancer mortality and the second leading cause of morbidity all over the world, accounting for 1,796,144 new deaths (18%) and 2,206,771 new cases (11.4%) in 2020 (1). From a pathological point of view, LC consists of a group of heterogeneous subtypes, in which the two major types are non-small cell lung cancer (NSCLC) and small cell lung cancer (SCLC) (2). As reported, approximately 85% of LC presented with the NSCLC, which can be further divided into lung adenocarcinoma (LUAD), lung squamous cell carcinoma (LUSC), large cell carcinoma, and mixed tissue carcinoma (3). Despite the advance in treatment methods and strategies, more than 50% of patients died within 1 year after being diagnosed with NSCLC and the 5-year survival rate remains poor (4). Fortunately, immunotherapy is becoming an alternative therapeutic approach for cancer patients, especially for NSCLC (5). At present, immunotherapy has become the first-line treatment of advanced NSCLC and for consolidation treatment of locally advanced NSCLC patients (6). Emerging lines of evidence suggested that the immune checkpoint inhibitors (ICIs) had brought promising changes in the treatment of advanced NSCLC patients and could prolong the progression-free survival (PFS) and overall survival (OS) (7). However, a considerable quantity of patients would not receive benefits from immunotherapy due to the tumor heterogeneity (8). Different from chemotherapy and targeted therapy, immunotherapy is not working on tumor cells themselves, but the tumor immune microenvironment (TME). However, fewer studies focusing on the correlation between biomarkers and tumor-infiltrating immune cells (TIICs), as well as the immune responses, are available. Therefore, discovering and identifying novel immune-related gene targets for NSCLC to improve the prognosis and promote progression of innovative therapy strategies remain crucial.

Interferon-induced protein 44-like (*IFI44L*) is a protein coding gene, belongs to the *IFI44* family and acts as a type I interferon-stimulated gene (ISG), and is composed of 452 amino acids and located on chromosome 1, area p31.1 (9). As reported, *IFI44L* may participate in numerous innate immune processes and could be induced by multiple viruses, including influenza virus and HIV-1 (10–12). In addition, the promoter methylation of *IFI44L* is a novel and appropriate diagnostic marker for systemic lupus erythematosus (SLE) patients, with a sensitivity of 88.571% and a specificity of 97.087% (13). Besides, *IFI44L* has been found to be upregulated after a wide range of viral infection and is involved in antiviral and anti-proliferative respiratory syncytial virus (RSV) infection procedure, suggesting that the *IFI44L* may serve as a promising biomarker of viral infection (14). Similarly, another research on the Japanese encephalitis virus (JEV) points that RIPK3 could inhibit the translation level of *IFI44L* and result in neuroinflammation or neuronal death, indicating that *IFI44L* played an important role in innate immune response (15). In addition to the abovementioned, recently studies reported that *IFI44L* was found to be involved in certain human tumorigenesis and progression. For instance, Huang et al. (16) demonstrated that *IFI44L* was downregulated in hepatocellular carcinoma and performed an anti-tumor effect through the met/Src signaling pathway. Wang et al. (17) found that miR-628-5p could promote migration and proliferation of osteosarcoma cells by regulating *IFI44L*, suggesting the potential position of *IFI44L* in diagnosis and treatment among osteosarcoma patients. However, the immunological role of *IFI44L* in tumor has not been discovered yet, especially in NSCLC. We hypothesize that *IFI44L* plays a vital role in the immune mechanism of NSCLC. This study was the first to investigate the immune implications of *IFI44L* in the development of NSCLC and its clinical significance.

Here, we systemically explored the immune implication of *IFI44L* in LUAD and LUSC through a bioinformatics method. Datasets available in this study were collected from The Cancer Genome Atlas (TCGA) and Gene Expression Omnibus (GEO) and utilized for subsequent analysis. We investigate the relationship between *IFI44L* and various tumor-infiltrating immune cells (TIICs) in TCGA-LUAD samples and TCGA-LUSC samples, respectively. Meanwhile, the immune-related pathways associated with *IFI44L* were also explored. Moreover, a number of immunomodulators that presented significant correlation with *IFI44L* were confirmed and used to construct the prognostic signature. Next, the prognostic efficiencies of the risk signatures were validated in the GSE72094 and GSE50081 dataset, respectively. A nomogram was established combining the TCGA-LUAD signature and corresponding clinical features to extend the practice value. Besides, functional experiments were performed to explore the effects of overexpression of *IFI44L* on proliferation, invasion, and migration in SPC-A-1 and NCI-H520 cells. The prognostic value and clinical relevance of *IFI44L* were also researched in the LC tissue microarray (TMA) cohort. Finally, the relationship between *IFI44L* expression and immunomodulators was verified in SPC-A-1 and NCI-H520 cell lines by the RT-qPCR method. We hope that this study would fill the gap of *IFI44L* in the LC domain, providing valuable information and laying the foundation for further research.

## Materials and Methods

### Data Collection and Processing

Public data included in this study were selected from the TCGA (The Cancer Genome Atlas) and GEO (Gene Expression Omnibus) database. The RNA-seq profiles and corresponding clinical information of TCGA-LUAD and TCGA-LUSC samples were downloaded from the UCSC website (https://xenabrowser.net). GSE72094 and GSE50081 were downloaded from the GEO database for the validation of LUAD signature and LUSC signature, respectively. Patients with complete transcriptome data and overall survival (OS) information were chosen for the subsequent analysis. As a result, a total of 497 TCGA-LUAD samples and 489 TCGA-LUSC samples were gathered as the training cohort. Besides, the GSE72094 dataset including 398 LUAD samples and the GSE50081 dataset including 43 LUSC samples were chosen to be the validation cohorts. The baseline information of these enrolled samples is shown in **Supplementary Table 1**. All the RNA-seq data were normalized by “scale” method for subsequent survival analysis.

### TIMER Database Analysis

TIMER (Tumor IMmune Estimation Resource, https://cistrome.shinyapps.io/timer/) served as a webtool for comprehensive analysis of TIICs in multiple cancers (18). In this study, we explored the relationship between *IFI44L* expression and six TIICs in the “Gene” module, including B cells, CD8^+^ T cells, CD4^+^ T cells, macrophages, neutrophils, and dendritic cells. In addition, the “SCNA” module was employed to investigate the correlation between infiltration levels with various somatic copy number alterations (deep deletion, arm-level deletion, diploid/normal, arm-level gain, and high amplification). The above analysis was performed in the TCGA-LUAD cohort and TCGA-LUSC cohort, respectively.

### CIBERSORT Analysis

To evaluate the difference of tumor-infiltrating immune cells (TIICs) in diverse groups, the CIBERSORT (Cell type Identification By Estimating Relative Subsets Of RNA Transcripts) method was employed to calculate the relative infiltration ratios of 22 kinds of TIICs among TCGA samples. As reported, the CIBERSORT algorithm could deconvolute the expression matrix of immune cell subtypes based on the principle of linear support vector regression (19). In this study, the transcriptome data were used to estimate the 22 kinds of TIICs in each enrolled sample, and samples with *p* > 0.05 were excluded. In addition, the selected samples were divided into two groups according to the median expression value of *IFI44L*, and the differences in lymphocytes between the two groups were compared.

### Gene Set Enrichment Analysis

Gene set enrichment analysis (GSEA) is a program that can distinguish whether a pre-defined set of genes shows statistical differences between two phenotypes based on computational method (20). In this section, we performed the GSEA analysis to explore the differential pathways and biology functions between the high-*IFI44L* group and the low-*IFI44L* group (version 4.0.3) in the TCGA-LUAD cohort and TCGA-LUSC cohort, respectively. The c2.cp.kegg.v7.1.symbols.gmt was selected as the reference gene sets. *p* < 0.05 was considered as statistically significant.

### ssGSEA Analysis

Single sample GSEA (ssGSEA) was a specific algorithm for quantitative immunocyte infiltration based on predetermined immunity marker genes. The 577 immunity marker genes were collected from previous research (21). By using this method, the landscape of 24 TIICs was evaluated in TCGA-NSCLC samples. Then, the association between *IFI44L* expression and these 24 TIICs were calculated by Spearman coefficients. Additionally, 70 immunomodulators were extracted from Charoentong’s study (22) and used to assess the Spearman correlation between *IFI44L* expression and these immunomodulators in TCGA-LUAD samples and TCGA-LUSC samples, respectively. *p* < 0.05 was considered as statistically significant.

### Protein–Protein Interaction and Enrichment Analysis

The STRING database (https://string-db.org/) served as an online tool for the exploration of interactions between proteins depending on previous publications (23). In this study, STRING 11.0 version was used to analyze the protein–protein interaction (PPI) network between *IFI44L* and its related immunomodulators in the TCGA-LUAD cohort. The confidence value was selected as “0.4”. Then, Gene Ontology (GO) and Kyoto Encyclopedia of Genes and Genomes (KEGG) enrichment analysis were employed to explore the biological functions and pathways of these immunomodulators. False discovery rate (FDR) < 0.05 was considered as statistically significant.

### Construction and Validation of Immunomodulator-Related Signatures

In order to clarify the prognostic value of *IFI44L*-related immunomodulators, the robust Cox regression models were utilized in the TCGA-LUAD cohort and the TCGA-LUSC cohort. The prognostic model complied with the Akaike information criterion (AIC) and the model that processed minimum AIC value was selected as the optimal result. The risk value of each sample was calculated as follows: risk score = Exp_1_*X_1_+ Exp_2_*X_2_+…+ Exp_n_*X_n_. Samples were divided into a high-risk group and a low-risk group according to the median cutoff value of risk score. Kaplan–Meier plots were conducted to compare the survival differences between the high-risk group and the low-risk group. Receiver operating characteristic (ROC) curves and concordance index (C-index) were employed to evaluate the prognostic model at 1 year, 3 years, and 5 years, respectively. Furthermore, the univariate and multivariate Cox regression was utilized to assess the independent prognostic efficacy of the two risk models in the TCGA-LUAD cohort and the TCGA-LUSC cohort. The clinical parameters included in the analysis were as follows: age (<65 vs. ≥65), gender (male vs. female), T (T1 vs. T2 vs. T3 vs. T4), N (N0 vs. N1), M (M0 vs. M1), and AJCC stage (stage I vs. stage II vs. stage III vs. stage IV). The final results were displayed by forest maps.

Next, another LUAD dataset (GSE72094) was chosen as the validation cohort for the TCGA-LUAD signature. As mentioned above, the GSE72094 cohort was divided into two groups by the same cutoff calculated in the TCGA-LUAD cohort. Kaplan–Meier survival plot was employed to compare the survival difference between the two groups and time-dependent ROC curves were performed to examine the specificity and sensitivity of the risk signature at 1 year, 3 years, and 5 years, respectively. Similarly, the univariate and multivariate Cox regression were conducted to evaluate whether the risk signature could act as an independent prognostic factor. Clinical information was as follows: age (<65 vs. ≥65), gender (male vs. female), smoking status (ever vs. never), and AJCC stage (stage I vs. stage II vs. stage III vs. stage IV). Similarly, GSE50081 cohort was performed to validate the TCGA-LUSC signature by the same method mentioned above.

### Establishment of Nomogram

Recently, nomogram has been increasingly used for predicting cancer-related prognosis. This method allows personalized estimates of the probability of recurrence, death, or drug compliance (24). Based on the prognostic model, this study established the nomogram in the TCGA-LUAD cohort by integrating the abovementioned clinical parameters to predict the OS probably at 1 year, 3 years, and 5 years. In addition, the calibration curves were performed to evaluate the fitness between actual survival status with observed survival status of the established nomogram through bootstrap methods (1,000 replicates). Last, the value of prognosis evaluation between stage, risk signature, and the nomogram was compared through ROC curves at 1 year, 3 years, and 5 years, respectively.

### Cell Culture

Human lung cancer cell lines SPC-A-1 and NCI-H520 were purchased from the cell banks of Type Culture Collection of the Chinese Academy of Sciences (Shanghai, China) and the American Type Culture Collection (ATCC, USA). Cells were cultured in RPMI-1640 medium containing 10% fetal bovine serum (Gibco, USA) at 5% CO_2_ and 37°C.

### Cell Transfection Experiment

The cDNA of human *IFI44L* gene was cloned into the mammalian expression vector pEGFP-N. The cells were evenly inoculated and cultured in a six-well plate for 24 h. Then, the target plasmid, the mixed system of ViaFect™ transfection reagent (Promega, USA), Opti-MEM medium, and complete medium were prepared to replace the medium in the six well plate. After 48 h of transfection, G418 solution was used to screen stably transfected cells.

### Cell Proliferation Assay

The cells were evenly inoculated on 96-well plates. Then, cell proliferation was respectively detected at 24 h, 48 h, and 72 h by CCK-8 assay. A mixed system of CCK-8 solution and RPMI-1640 complete medium was prepared according to the instructions of CCK-8 Kit (Dojindo, Japan). Finally, the OD value was determined by measuring the absorbance on 450 nm. All tests were conducted at least 3 times and repeated 3 times each time.

### Scratch Healing Experiment

The cells were evenly seeded in a six-well plate and then cell transfection was performed 24 h later. After 24 h of transfection, a sterile toothpick was used to scratch each hole of the six-well plate. At 0 h and 48 h, the inverted microscope was used to observe and photograph the scratched cells at the corresponding positions. Finally, the cell migration distance was statistical analyzed. All measurements are performed at least 3 times, and each time is repeated 3 times.

### Transwell Assay

Approximately 2 × 10^4^ cells were seeded in a transwell chamber (Corning, USA). Transwell chamber with and without matrix gel was used to detect cell invasion and cell migration, respectively. After culturing for 24 h under 5% CO_2_ and 37°C, the cells were fixed with 4% paraformaldehyde for 30 min at room temperature, stained with crystal violet staining solution for 10 min, and photographed under an inverted microscope. Finally, 5 different fields were randomly selected for statistical analysis. All tests should be performed at least 3 times, each time repeated 3 times.

### RT-qPCR

Total RNA was extracted from cells using Trizol (Invitrogen, USA), which was further reversely transcribed into cDNA using the GoScript™ reverse transcription system (Promega, USA) after assessing the concentration and the purity. Then, GoTaq^®^ qPCR Master Mix Kit (Promega) was used for RT-qPCR, and β-actin served as the internal control for all PCR reactions. All data from the calibration samples were analyzed by the 2^−ΔΔCt^ method and all experiments were performed at least in triplicate. Primers are listed in **Supplementary Table 2**.

### Immunohistochemistry

TMA with 97 NSCLC tumor tissues was obtained from Shanghai Outdo Biotech Co, LTD of China (TMA number: HLugA180Su07). These patients with NSCLC did not receive any preoperative anti-cancer treatment before surgery. Moreover, the diagnosis of NSCLC is confirmed based on pathological evidence. Written informed consents of all patients were obtained before the study. The use of clinical specimens for research purposes has been approved by the Research Ethics Committee of Shanghai Outdo Biotech Co, LTD. The tumor clinical stages and differentiation grades were classified according to the 7th American Joint Committee on Cancer (AJCC) TNM classification. IFI44L antibody (NBP1-57838, NOVUS, USA) was used to perform immunohistochemical staining of the TMA chips as described previously (16). IFI44L protein expression level was calculated as the product of the percentage of the staining intensity between positively stained cells. A final staining score greater than or equal to eight was defined as high expression.

### Statistical Analysis

All the statistical analyses were performed by R 4.0.3 (http://www.R-project.org) using the appropriate packages. Wilcoxon test was used to compare the differences of immune infiltration levels between two groups. The correlation between *IFI44L* and TIICs and immunomodulators were evaluated by Spearman coefficient. The log-rank test was used to calculate the statistical significance of survival rate between different risk groups. ROC curves were used to evaluate the sensitivity and specificity of the established signature. Univariate and multivariate Cox regression were performed to explore the significant prognosis factors. For categorical variables, *χ* ^2^ test or Fisher exact test was employed to analyze the correlation between diverse variables. Measurement data were compared by Student’s *t*-test between different groups. Unless otherwise specified, *p* < 0.05 was considered as statistically significant.

## Results

### Flow Chart of This Study

**Figure 1:**
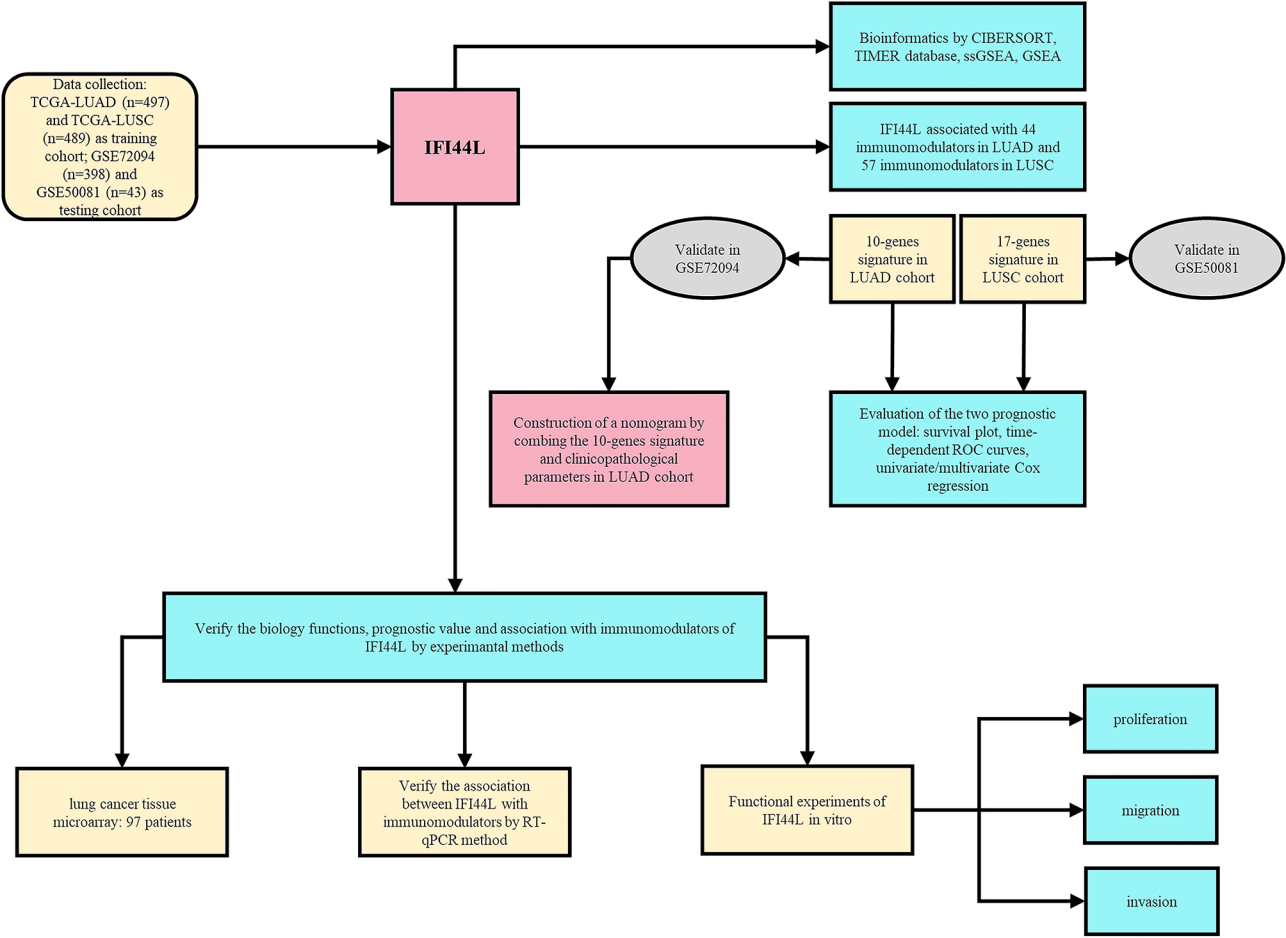
Flowchart of this study.

As shown in **Figure 1**, our research was designed and performed according to this technical process. Firstly, the immune implication of *IFI44L* was explored through various bioinformatics methods, including TIMER database analysis, CIBERSORT analysis, and GSEA and ssGSEA analysis. In addition, the *IFI44L*-associated immunomodulators were selected and applied to construct the prognostic signature in the TCGA-LUAD cohort and the TCGA-LUSC cohort, respectively. GSE72094 and GSE50081 were used to validate these two signatures mentioned above. Besides, by combining the LUAD signature and clinicopathological parameters, we established a nomogram in the TCGA-LUAD cohort and evaluated its prognostic efficacy by calibration curves and time-dependent ROC curves. Finally, a TMA cohort was obtained to address the association between the translation level of *IFI44L* with clinical relevance and survival outcome. Meanwhile, *in vitro* experiments were implied to verify the biological functions and relationship with multiple immunomodulators of *IFI44L* in *IFI44L*-overexpressed cell models.

### *IFI44L* Showed Significant Correlation With Various TIICs in NSCLC

**Figure 2:**
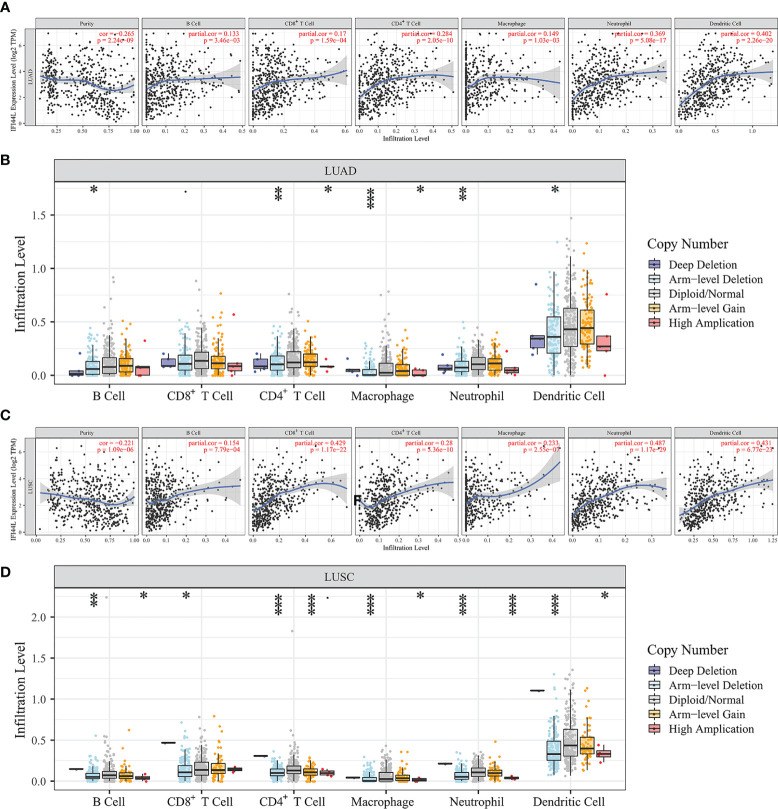
Exploration of the immune association of *IFI44L* in LUAD and LUSC samples through TIMER database. **(A, C)** Relevance between *IFI44L* and six TIICs in LUAD samples **(A)** and LUSC samples **(C)**. **(B, D)** Comparison of infiltration levels among tumor samples with different CNV types of *IFI44L* in LUAD samples **(B)** and LUSC samples **(D)**. TIMER, Tumor IMmune Estimation Resource; CNV, copy number variation; TCGA, The Cancer Genome Atlas; LUAD, lung adenocarcinoma; LUSC, lung squamous cell carcinoma; TIICs, tumor-infiltrating immune cells; *p < 0.05, **p < 0.01, ***p < 0.001.

To explore the association between *IFI44L* expression and TIICs, we searched the TIMER database to complete this assignment. The results indicated that *IFI44L* showed significant correlation with the B cell (*r* = 0.133, *p* = 0.003), CD8^+^ T cell (*r* = 0.17, *p* < 0.001), CD4^+^ T cell (*r* = 0.284, *p* < 0.001), macrophage (*r* = 0.149, *p* = 0.001), neutrophil (*r* = 0.369, *p* < 0.001), and dendritic cell (*r* = 0.402, *p* < 0.001, **Figure 2A**) in LUAD samples. In addition, the infiltration levels and copy number variation (CNV) were further explored in “SCNA” module. From the results, we know that with the arm-level deletion of *IFI44L*, the infiltration level of B cell (*p* = 0.032), CD4^+^ T cell (*p* = 0.003), macrophage (*p* < 0.001), neutrophil (*p* = 0.009), and dendritic cell (*p* = 0.021, **Figure 2B**) was significant decreased. Moreover, *IFI44L* presented similar correlation with B cell (*r* = 0.154, *p* < 0.001), CD8^+^ T cell (*r* = 0.429, *p* < 0.001), CD4^+^ T cell (*r* = 0.28, *p* < 0.001), macrophage (*r* = 0.233, *p* < 0.001), neutrophil (*r* = 0.487, *p* < 0.001), and dendritic cell (*r* = 0.431, *p* < 0.001, **Figure 2C**) in LUSC samples. Consistently, the CNV analysis indicated that B cell (*p* = 0.005), CD8^+^ T cell (*p* = 0.045), CD4^+^ T cell (*p* < 0.001), macrophage (*p* < 0.001), neutrophil (*p* < 0.001), and dendritic cell (*p* < 0.001, **Figure 2D**) showed lower infiltration level with arm-level deletion of *IFI44L*.

In order to further assess the relevance between *IFI44L* expression and various TIICs in NSCLC, we extracted and processed characteristic gene expression profiles by the CIBERSORT method. By removing the samples with *p* > 0.05, 448 tumor samples in the TCGA-LUAD cohort and 442 tumor samples in the TCGA-LUSC cohort were collected for subsequent analysis. Firstly, we compared the difference of TIICs between the high-*IFI44L* group and the low-*IFI44L* group based on the median cutoff value of *IFI44L* expression. Notably, when compared with the low-*IFI44L* group, the proportion of activated CD4 memory T cells, regulatory T cells (Tregs), macrophage M1, and activated dendritic cells were significantly increased while the plasma cells were decreased in the high-*IFI44L* group in the TCGA-LUAD cohort (*p* < 0.05, **Supplementary Figure 1A**). Besides, in the TCGA-LUSC cohort, the CD8 T cells, activated CD4 memory T cells, resting NK cells, and macrophage M1 presented a higher proportion in the high-*IFI44L* group, while the plasma cells, macrophages M0, and resting dendritic cells showed a lower proportion (*p* < 0.05, **Supplementary Figure 1B**). These results indicated that *IFI44L* performed an obvious association with various infiltrating lymphocytes and is worthy of further research.

In addition, the landscape of 22 TIICs between 57 adjacent normal samples and 448 tumor samples was also conducted in the TCGA-LUAD cohort. The results showed that the proportions of memory B cells, plasma cells, T cells CD4 memory activated, follicular helper T cells, Tregs, macrophages M1, and dendritic resting cells were significantly increased (*p* < 0.05) in tumor samples, while the CD4 memory resting T cells, resting NK cells, monocytes, macrophages M0, macrophages M2, activated dendritic cells, resting mast cells, eosinophils, and neutrophils were significantly decreased (*p* < 0.05, **Supplementary Figure 2A**). A similar result was presented in the TCGA-LUSC cohort (**Supplementary Figure 2B**).

### *IFI44L* Participated in Multiple Immune/Cancer-Related Pathways in NSCLC Samples

In order to further analyze the potential functions of *IFI44L* in NSCLC patients, the GSEA was performed to explore the candidate pathways between the high-*IFI44L* group and the low-*IFI44L* group in the TCGA-LUAD cohort and TCGA-LUSC cohort, respectively. The results showed that *IFI44L* associated with multiple immune/cancer-related signaling pathways in the TCGA-LUAD cohort, including T-cell receptor signaling pathway (NES = 1.98, *p* = 0.002), B-cell receptor signaling pathway (NES = 1.88, *p* = 0.006), chemokine signaling pathway (NES = 2.06, *p* < 0.001), FcγR-mediated phagocytosis (NES = 1.82, *p* = 0.004), cytokine–cytokine receptor interaction (NES = 2.09, *p* < 0.001), natural killer cell-mediated cytotoxicity (NES = 2.29, *p* < 0.001), FcϵRI signaling pathway (NES = 1.68, *p* = 0.023), P53 signaling pathway (NES = 1.65, *p* = 0.018), NOD-like receptor signaling pathway (NES = 2.27, *p* < 0.001), and JAK/STAT signaling pathway (NES = 2.29, *p* < 0.001, **Supplementary Figure 3A**). Similar results were observed in the TCGA-LUSC cohort (**Supplementary Figure 3B**), including T-cell receptor signaling pathway (NES = 2.11, *p* < 0.001), B-cell receptor signaling pathway (NES = 1.82, *p* = 0.008), cytokine–cytokine receptor interaction (NES = 2.46, *p* < 0.001), antigen processing and presentation (NES = 2.39, *p* < 0.001), natural killer cell-mediated cytotoxicity (NES = 2.38, *p* < 0.001), JAK/STAT signaling pathway (NES = 2.33, *p* < 0.001), apoptosis (NES = 2.24, *p* < 0.001), chemokine signaling pathway (NES = 2.24, *p* < 0.001), FcϵRI signaling pathway (NES = 1.73, *p* = 0.008), and intestinal immune network for IgA production (NES = 2.20, *p* < 0.001). These results strongly suggested that *IFI44L* may participate in multiple immune/cancer-related pathways in NSCLC patients and thus played a vital role in tumorigenesis and progression.

### The Statistical Association Between *IFI44L* Expression and Immunomodulators in TCGA Samples

**Figure 3:**
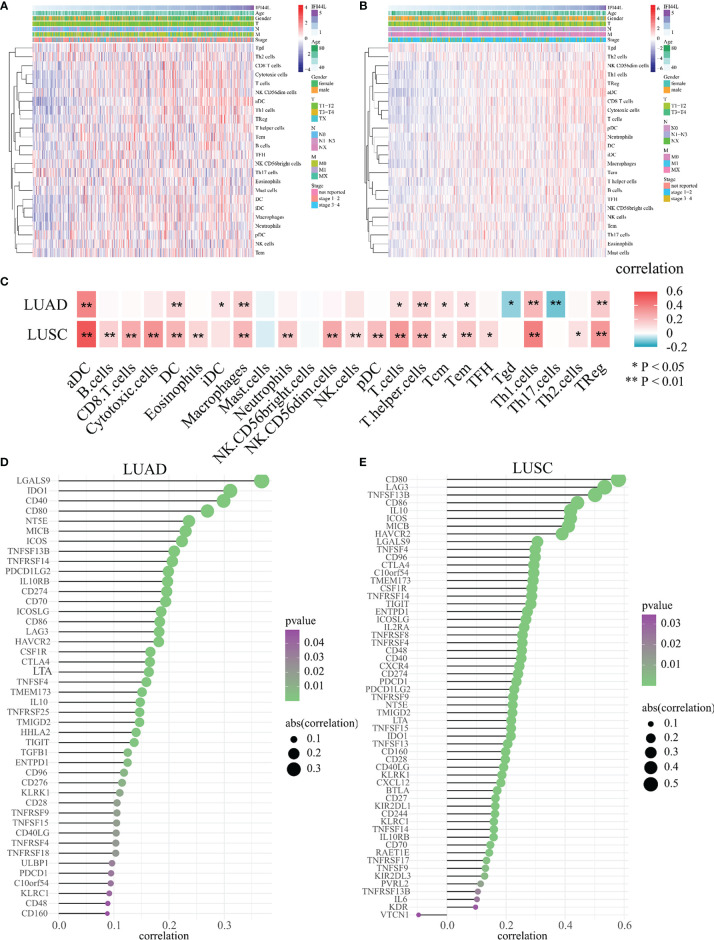
Evaluation of the association between *IFI44L* transcription level with 24 TIICs and 70 immunomodulators in TCGA-NSCLC samples. **(A, B)** Infiltration proportions of 24 TIICs were calculated by the ssGSEA method in each sample and presented by heatmap among TCGA-LUAD samples **(A)** and TCGA-LUSC samples **(B)**. **(C)** Correlation heatmap showed the significant relevance between *IFI44L* expression and 24 TIICs in TCGA-LUAD samples and TCGA-LUSC samples. **(D, E)** Identification and analysis of immunomodulators associated with the *IFI44L* gene in TCGA-LUAD samples **(D)** and TCGA-LUSC samples **(E)**. Immunomodulators that possess the statistically significant Spearman coefficients with *IFI44L* are shown in the graph. TIICs, tumor-infiltrating immune cells; TCGA, The Cancer Genome Atlas; LUAD, lung adenocarcinoma; LUSC, lung squamous cell carcinoma; *p < 0.05, **p < 0.01.

Next, for the purpose of further evaluating the impact of *IFI44L* on the tumor microenvironment (TME), we analyzed the relationship between *IFI44L* and 24 TIICs through the ssGSEA method in TCGA samples. The heatmap presented the correlation between *IFI44L* expression, infiltration proportions of 24 TIICs, and clinical parameters in TCGA-LUAD samples (**Figure 3A**) and TCGA-LUSC samples (**Figure 3B**), respectively. From the results, we know that *IFI44L* showed a positive association with various TIICs, whether in TCGA-LUAD samples or TCGA-LUSC samples. Then, the Spearman correlation coefficients were calculated between *IFI44L* expression and immune infiltration level. The results showed that some TIICs were either negatively or positively associated with the transcription level of *IFI44L* in LUAD and LUSC samples (**Figure 3C**). For details, 12/24 TIICs showed obvious correlation (*p* < 0.05) with *IFI44L* in the TCGA-LUAD cohort, including activated dendritic cells (aDC), dendritic cells (DC), immature dendritic cells (iDC), macrophages, T cells, T helper cells, central memory T cells (Tcm), effector memory T cells (Tem), gamma delta T cells (Tgd), type 1 T helper cells (Th1 cells), type 17 T helper cells (Th17 cells), and Treg. More importantly, 19/24 TIICs presented significant coefficients (*p* < 0.05) with *IFI44L* in the TCGA-LUSC cohort, including aDC, B cells, CD8 T cells, cytotoxic cells, DC, eosinophils, macrophages, neutrophils, CD56dim natural killer cells (NK CD56dim cells), NK cells, plasmacytoid dendritic cells (pDC), T cells, T helper cells, Tcm, Tem, T follicular helper cells (TFH), Th1 cells, Th2 cells, and Treg. In order to further analyze the relationship between *IFI44L* and immunity, we explored the Spearman coefficients between *IFI44L* and 70 immunomodulators among TCGA samples. As a result, a total of 44 immunomodulators were found to be significantly correlated with *IFI44L* in the TCGA-LUAD (**Figure 3D**, *p* < 0.05) cohort and 57 immunomodulators were found to be significantly correlated with *IFI44L* in the TCGA-LUSC (**Figure 3E**, *p* < 0.05) cohort. Correlation plots between *IFI44L* and each immunomodulator are shown in **Supplementary Figure 4** (TCGA-LUAD) and **Supplementary Figure 5** (TCGA-LUSC).

In addition, the 44 immunomodulators in the TCGA-LUAD cohort were further analyzed by the STRING database. A total of 45 nodes and 740 edges were gathered in the network (**Supplementary Figure 6A**). Finally, GO and KEGG enrichment analysis was performed to explore the potential functions of these immunomodulators. KEGG enrichment analysis suggested that these genes mainly participate in the cytokine–cytokine receptor interaction, T-cell receptor signaling pathway, natural killer cell-mediated cytotoxicity, NF-κB signaling pathway, PD-L1 expression and PD-L1 checkpoint pathway in cancer, etc. (**Supplementary Figure 6B**). GO enrichment analysis showed that these immunomodulators mainly associated with immune-related biological functions (**Supplementary Figure 6C**).

### Prognostic Implication of *IFI44L*-Related Immunomodulators in TCGA-NSCLC Samples

**Figure 4:**
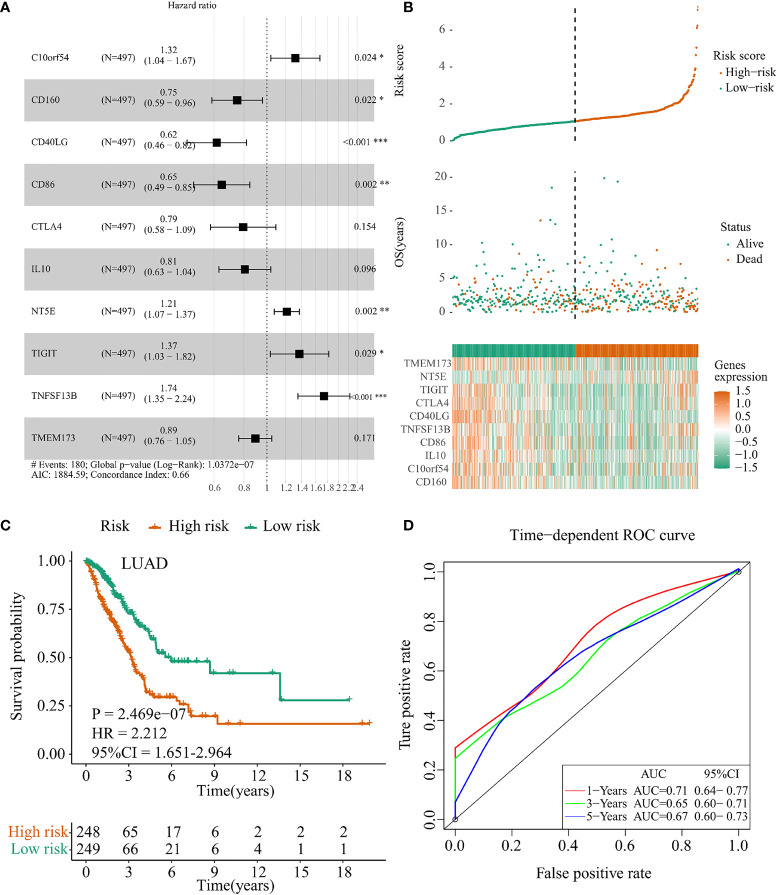
Construction of the *IFI44L*-related immunomodulators signature in TCGA-LUAD cohort. **(A)** Development of the prognostic signature based on 44 *IFI44L*-associated immunomodulators through multivariate stepwise Cox analysis, and the results are shown in a forest plot. **(B)** Distribution of the risk score, survival status, and expression profiling of immunomodulators integrated in this signature. **(C)** The cohort was separated by median cutoff value of risk score, and the difference in survival was performed using a Kaplan–Meier plot. **(D)** Time-dependent ROC curves at 1 year, 3 years, and 5 years for evaluating the specificity and sensitivity of this risk signature. TCGA, The Cancer Genome Atlas; LUAD, lung adenocarcinoma; ROC, receiver operating characteristic. *p < 0.05, **p < 0.01, ***p < 0.001.

In order to investigate the prognostic value of *IFI44L*-related immunomodulators in TCGA-LUAD and TCGA-LUSC patients, we performed multivariate stepwise Cox regression depending on the immunomodulators obtained above and established the prognostic models, respectively. As a result, a 10-immunomodulator signature (*TMEM173*, *NT5E*, *TIGIT*, *CTLA4*, *CD40LG*, *TNFSF13B*, *CD86*, *IL10*, *C10orf54*, and *CD160*) was constructed in the TCGA-LUAD cohort with the best AIC = 1,884.59 (**Figure 4A**). The C-index of this risk signature was 0.661. Functions and risk coefficients of these chosen immunomodulators are listed in **Supplementary Table 3**. The risk score was calculated by adding the product of each gene expression value with the corresponding risk coefficient. The median risk score (risk score = 1.062) was selected as the optimal cutoff value and used to divide the TCGA-LUAD patients into a high-risk group and a low-risk group. **Figure 4B** shows the distribution of risk score, survival status, and immunomodulator expression profiling in the TCGA-LUAD cohort. Kaplan–Meier plot showed that patients in the low-risk group had significantly longer OS times than those in the high-risk group (*p* < 0.001, HR = 2.212, 95% CI = 1.651–2.964, **Figure 4C**). The area under curve (AUC) values and 95% confidence interval (CI) at 1 year, 3 years, and 5 years were 0.71 (0.64–0.77), 0.65 (0.60–0.71), and 0.67 (0.60–0.73), respectively (**Figure 4D**). In addition, to clarify whether the LUAD signature could conduct good performance among other subtype of NSCLC samples, we examined the prognostic efficacy of the TCGA-LUAD signature in the TCGA-LUSC cohort. Regrettably, the result was not very satisfactory (**Supplementary Figure 7**, *p* = 0.705). This phenomenon indicated that the TCGA-LUAD signature had a certain degree of specificity.

**Figure 5:**
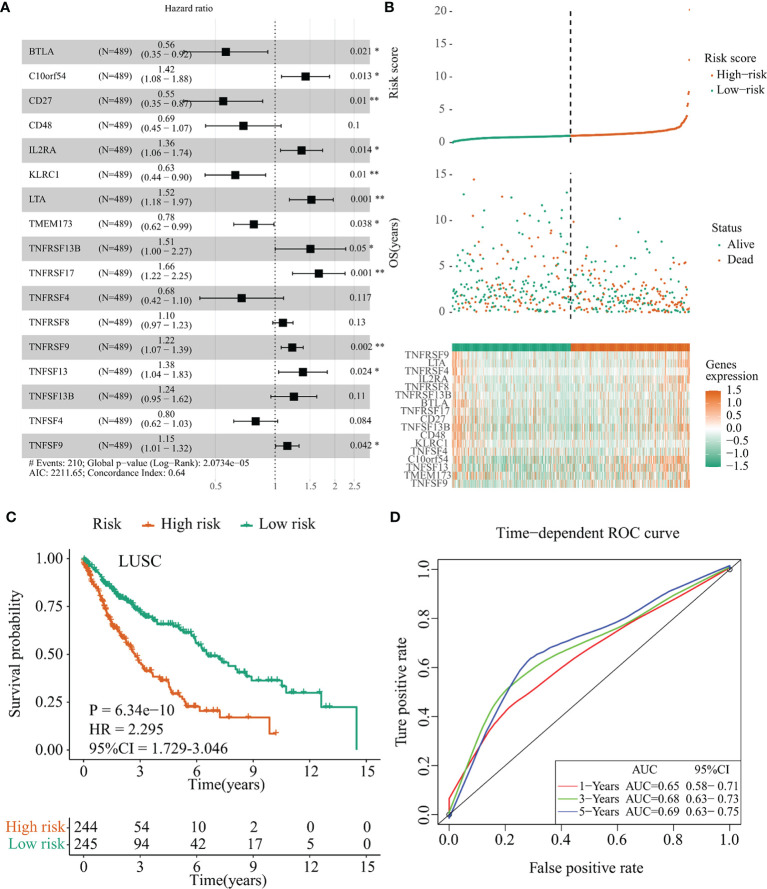
Establishment of the *IFI44L*-related immunomodulator signature in the TCGA-LUSC cohort. **(A)** Development of the prognostic signature based on 57 *IFI44L*-associated immunomodulators through multivariate stepwise Cox analysis, and the results are shown in a forest plot. **(B)** Distribution of the risk score, survival status, and expression profiling of immunomodulators integrated in this signature. **(C)** The cohort was separated by median cutoff value of risk score, and the difference in survival was performed using a Kaplan–Meier plot. **(D)** Time-dependent ROC curves at 1 year, 3 years, and 5 years for evaluating the specificity and sensitivity of this risk signature. TCGA, The Cancer Genome Atlas; LUSC, lung squamous cell carcinoma; ROC, receiver operating characteristic. *p < 0.05, **p < 0.01.

Similarly, a 17-immunomodulator signature (*TNFRSF9*, *LTA*, *TNFRSF4*, *IL2RA*, *TNFRSF8*, *TNFRSF13B*, *BTLA*, *TNFRSF17*, *CD27*, *TNFSF13B*, *CD48*, *KLRC1*, *TNFSF4*, *C10orf54*, *TNFSF13*, *TMEM173*, and *TNFSF9*) was established in the TCGA-LUSC cohort through the same method. The optimal AIC of this 17-immunomodulator signature was 2,211.65 and the C-index was 0.640 (**Figure 5A**). **Supplementary Table 4** summarized the functions and risk coefficients of these 17 immunomodulators. As abovementioned, the TCGA-LUSC cohort was separated by the median cutoff value of risk score (risk score = 1.03). Distribution of risk score, survival status, and expression of these 17 immunomodulators are presented in **Figure 5B**. Survival analysis indicated that patients in the low-risk group had significantly longer OS times than those in the high-risk group (*p* < 0.001, HR = 2.295, 95% CI = 1.729–3.046, **Figure 5C**). The AUC values and 95% CI at 1 year, 3 years, and 5 years were 0.65 (0.58–0.71), 0.68 (0.63–0.73), and 0.69 (0.63–0.75), respectively (**Figure 5D**).

### The Risk Models Could Act as Independent Prognostic Factors in TCGA-NSCLC Samples

**Figure 6:**
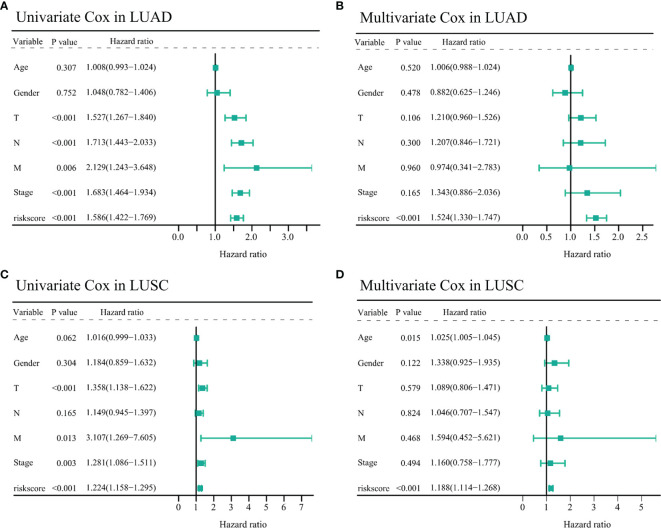
Assessing the prognostic values of the two *IFI44L*-related immunomodulator signatures in TCGA-NSCLC cohorts. **(A, B)** Univariate **(A)** and multivariate **(B)** Cox regression analysis of the risk score in the TCGA-LUAD cohort. **(C, D)** Univariate **(C)** and multivariate **(D)** Cox regression analysis of the risk score in the TCGA-LUSC cohort. TCGA, The Cancer Genome Atlas; NSCLC, non-small cell lung carcinoma; LUAD, lung adenocarcinoma; LUSC, lung squamous cell carcinoma.

To identify whether the risk models could serve as independent prognostic factors in TCGA-NSCLC samples, we performed univariate and multivariate Cox regression to confirm this hypothesis. In the TCGA-LUAD cohort, univariate analysis showed that T (HR = 1.527, 95% CI = 1.267–1.840, *p* < 0.001), N (HR = 1.713, 95% CI = 1.443–2.033, *p* < 0.001), M (HR = 2.129, 95% CI = 1.243–3.648, *p* = 0.006), stage (HR = 1.683, 95% CI = 1.464–1.934, *p* < 0.001), and risk score (HR = 1.586, 95% CI = 1.422–1.769, *p* < 0.001, **Figure 6A**) were significantly associated with OS outcome. Besides, the multivariate Cox regression suggested that the risk score remained an independent prognostic factor for TCGA-LUAD patients (HR = 1.524, 95% CI = 1.330–1.747, *p* < 0.001, **Figure 6B**) after adjustment for age, T, N, M, and stage.

Moreover, in the TCGA-LUSC cohort, clinical parameters including the T (HR = 1.358, 95% CI = 1.138–1.622, *p* < 0.001), M (HR = 3.107, 95% CI = 1.269–7.605, *p* = 0.013), stage (HR = 1.281, 95% CI = 1.086–1.511, *p* = 0.003), and risk score (HR = 1.224, 95% CI = 1.158–1.295, *p* < 0.001) showed significant correlation with OS through univariate Cox analysis (**Figure 6C**). Similar to the abovementioned, the multivariate Cox regression indicated that age (HR = 1.025, 95% CI = 1.005–1.045, *p* = 0.015) and risk score (HR = 1.188, 95% CI = 1.114–1.268, *p* < 0.001) may act as independent prognostic factors for TCGA-LUSC patients (**Figure 6D**). All these results indicated that the *IFI44L*-related immunomodulator signatures presented significant correlation with the prognosis of TCGA-NSCLC patients.

### Validation of the TCGA-LUAD and TCGA-LUSC Signature in the GSE72094 Dataset and the GSE50081 Dataset, Respectively

**Figure 7:**
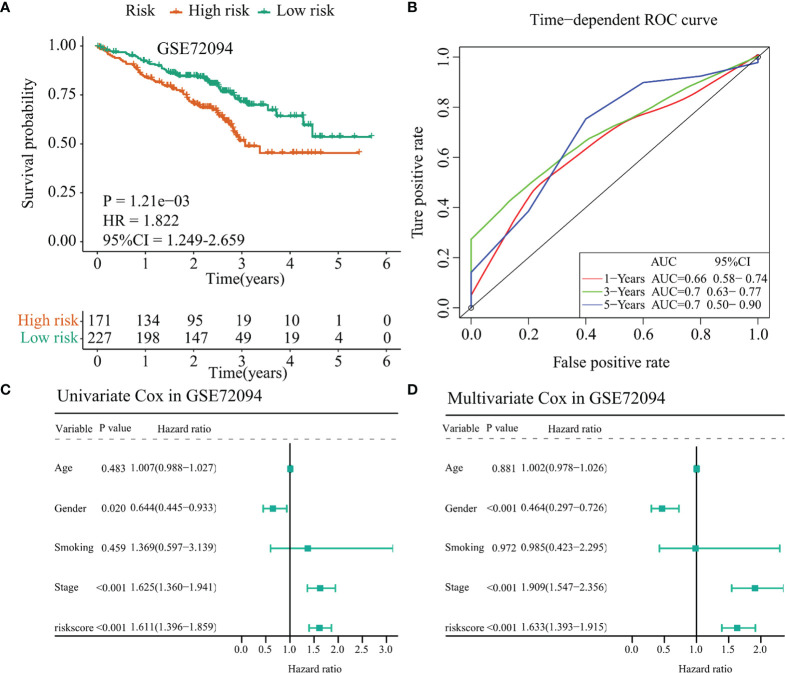
Validation of the TCGA-LUAD signature in the GSE72094 dataset. **(A)** Kaplan–Meier curve presented the survival difference between the high-risk group and the low-risk group. **(B)** Time-dependent ROC curves at 1 year, 3 years, and 5 years. **(C)** Univariate Cox regression analysis regarding the risk score and other clinical parameters. **(D)** Multivariate Cox regression analysis about the risk score and else clinical parameters. TCGA, The Cancer Genome Atlas; LUAD, lung adenocarcinoma.

The GSE72094 dataset was selected as a validation cohort for accessing the performance of the 10-immunomodulator signature constructed in the TCGA-LUAD cohort. Patients in GSE72094 were divided into a high-risk group and a low-risk group by the same cutoff value (risk score = 1.062). The C-index of this risk signature in GSE72094 was 0.615. Survival analysis showed that patients in the high-risk group had less OS time (*p* = 0.001, HR = 1.822, 95% CI = 1.249–2.659, **Figure 7A**) compared with the low-risk group. Time-dependent ROC curves presented that the AUC values for this 10-immunomodulator signature at 1 year, 3 years, and 5 years were 0.66 (0.58–0.74), 0.7 (0.63–0.77), and 0.7 (0.50–0.90), respectively (**Figure 7B**). Furthermore, univariate Cox regression showed that gender (HR = 0.644, 95% CI = 0.445–0.933, *p* = 0.020), stage (HR = 1.625, 95% CI = 1.360–1.941, *p* < 0.001), and risk score (HR = 1.611, 95% CI = 1.396–1.859, *p* < 0.001) were notably associated with OS outcome (**Figure 7C**). It is worth noting that the risk score may act as an independent prognostic factor after multivariate Cox regression analysis (HR = 1.633, 95% CI = 1.393–1.915, *p* < 0.001, **Figure 7D**). Collectively, the above results indicated that the 10-immunomodulator signature displayed a good performance in predicting OS status among LUAD patients.

**Figure 8:**
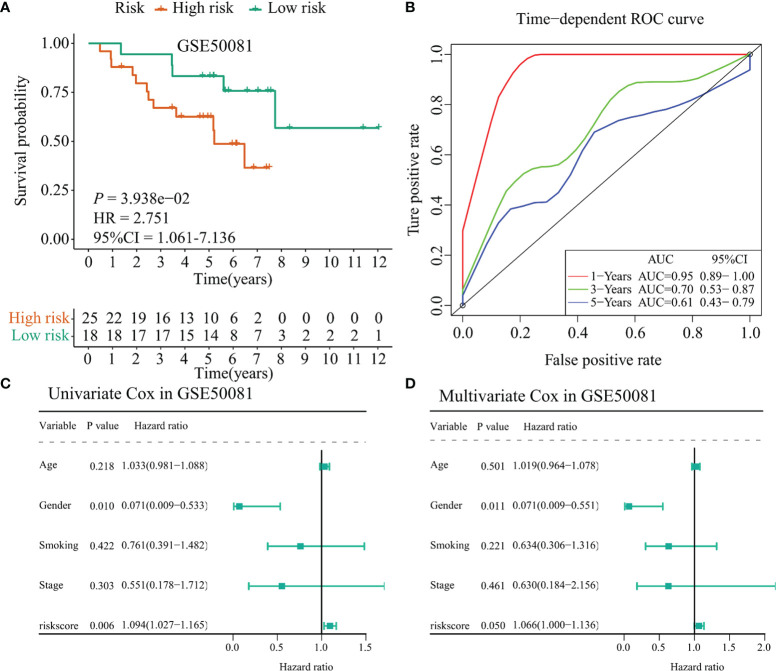
Validation of the TCGA-LUSC signature in the GSE50081 dataset. **(A)** Kaplan–Meier curve presented the survival difference between high-risk group and low-risk group. **(B)** Time-dependent ROC curves at 1 year, 3 years, and 5 years. **(C)** Univariate Cox regression analysis regarding the risk score and other clinical parameters. **(D)** Multivariate Cox regression analysis about the risk score and else clinical parameters. TCGA: The Cancer Genome Atlas; LUSC: lung squamous cell carcinoma.

Besides, we selected the GSE50081 dataset to verify the prognostic value of the 17-immunomodulator signature constructed in the TCGA-LUSC cohort. A total of 43 LUSC samples were enrolled in this section. Using the same method as mentioned above, the 43 LUSC samples were divided into a high-risk group and a low-risk group according to the cutoff value of risk score calculated in the TCGA-LUSC cohort. Notably, the high-risk group had even worse outcome compared with the low-risk group (*p* = 0.039, HR = 2.751, 95% CI = 1.061–7.136, **Figure 8A**). Time-dependent ROC curves showed that the 1-year, 3-year, and 5-year AUC values were 0.95 (0.89–1.00), 0.70 (0.53–0.87), and 0.61 (0.43–0.79), respectively (**Figure 8B**). In addition, the univariate Cox regression showed that gender (HR = 0.071, 95% CI = 0.009–0.533, *p* = 0.010) and risk score (HR = 1.094, 95% CI = 1.027–1.165, *p* = 0.006) were significantly associated with OS status among 43 LUSC samples (**Figure 8C**). Interestingly, multivariate Cox regression indicated that the risk score may act as an independent prognostic factor in LUSC samples (HR = 1.066, 95% CI = 1.000–1.136, *p* = 0.050, **Figure 8D**), even though the specific *p*-value did not match the statistical significance. Collectively, the above results indicated that the 17-immunomodulator signature both displayed good performances in predicting OS status among LUSC patients.

### Establishment and Evaluation of the Nomogram Based on the TCGA-LUAD Signature and Clinicopathological Parameters

**Figure 9:**
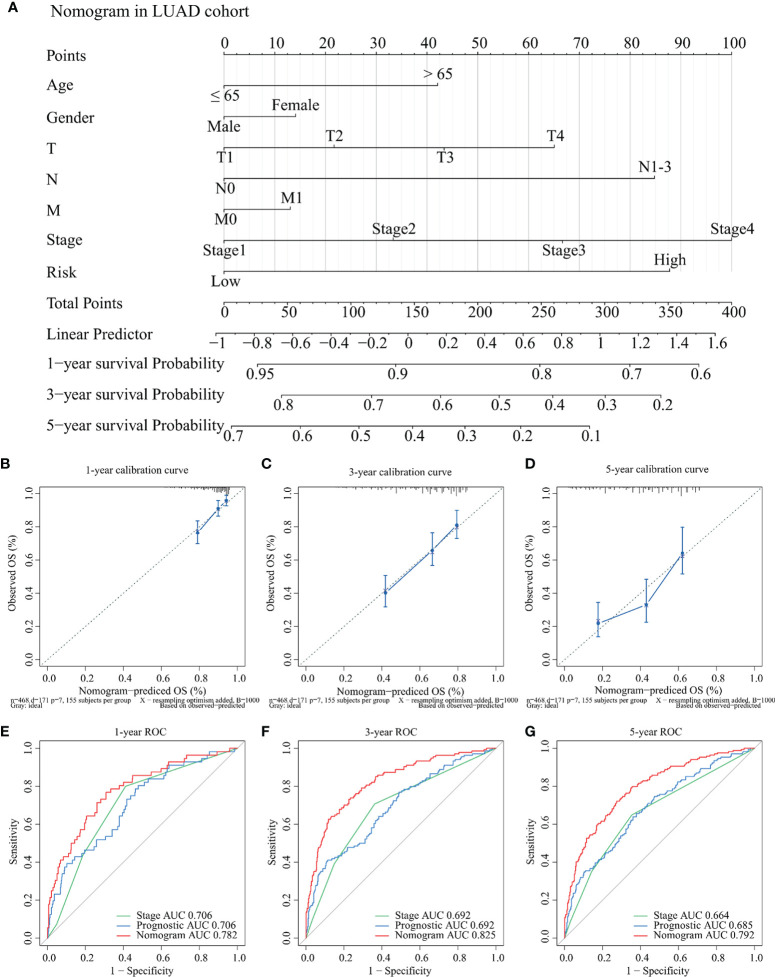
Development and evaluation of the nomogram for OS among TCGA-LUAD patients. **(A)** A nomogram was established for predicting the OS at 1 year, 3 years, and 5 years by weighting age, gender, T, N, M, stage, and risk score, respectively. **(B–D)** Calibration curves about the nomogram in 1 year **(B)**, 3 years **(C)**, and 5 years **(D)**. The *x*-axis represents predicting OS outcome and the *y*-axis represents actual OS outcome. **(E–G)** Comparison of ROC curves between stage, risk score, and nomogram at 1 year **(E)**, 3 years **(F)**, and 5 years **(G)**, respectively. OS: overall survival; TCGA: The Cancer Genome Atlas; LUAD: lung adenocarcinoma; ROC: receiver operating characteristic.

To further clarify the application value of the TCGA-LUAD risk model in clinical practice, a nomogram was constructed for predicting the OS probabilities at 1 year, 3 years, and 5 years by combining the risk model with clinicopathological parameters (age, gender, T, N, M, and stage) in the TCGA-LUAD cohort (**Figure 9A**). Notably, the C-index of the nomogram was 0.775. Calibration curves suggested that the nomogram performed good fitness between predicting OS and observed OS at 1 year, 3 years, and 5 years (**Figures 9B–D**). Finally, the AUC values of nomogram, stage, and risk model were also compared. Detailed comparison results are presented in **Table 1**. Obviously, the nomogram showed better performance than stage model and risk model (*p* < 0.05), and the corresponding AUCs were 0.782 at 1 year, 0.825 at 3 years, and 0.792 at 5 years (**Figures 9E–G**).

**Table 1:** Comparison of the AUC values between stage model, prognostic model, and the nomogram.

Model	Stage model	Prognostic model	Nomogram	Stage vs. Prognostic	Nomogram vs. Stage	Nomogram vs. Prognostic
1-year AUC	0.706 (0.641–0.771)	0.706 (0.633–0.778)	0.782 (0.715–0.849)	0.971	0.007^**^	0.04*
3-year AUC	0.692 (0.642–0.741)	0.692 (0.639–0.745)	0.825 (0.782–0.867)	0.990	<0.001^***^	<0.001^***^
5-year AUC	0.664 (0.617–0.712)	0.685 (0.635–0.734)	0.792 (0.750–0.833)	0.544	<0.001^***^	<0.001^***^

AUC, areas under the curve; ^*^p < 0.05, ^**^p < 0.01, ^***^p < 0.001.

### Overexpression of *IFI44L* Inhibited Cell Proliferation, Migration, and Invasion in LUSC and LUAD

**Figure 10:**
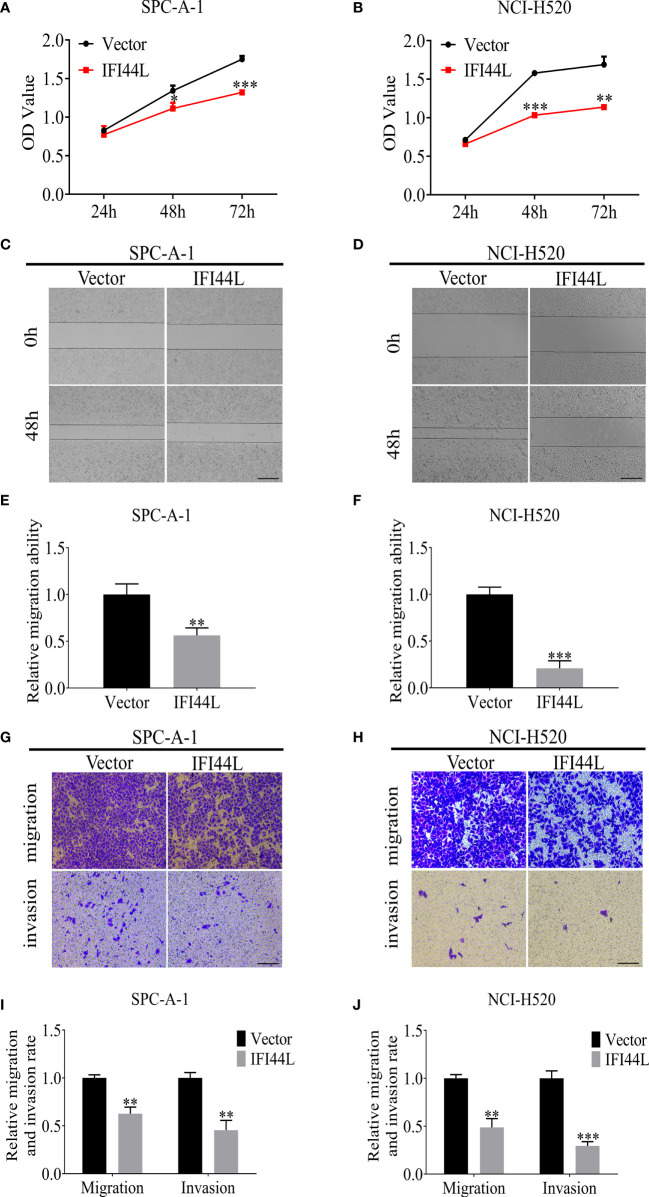
Overexpression of *IFI44L* gene inhibited the proliferation, migration, and invasion of NSCLC cells. **(A, B)** CCK-8 assay was used to detect the effect of *IFI44L* overexpression on NSCLC cell growth. **(C–F)** The cell migration was detected after overexpression of *IFI44L* gene by scratch healing tests. Bar equals 100 µm. **(G–J)** Overexpression of *IFI44L* inhibited the migration and invasion of NSCLC cells by transwell assay. Bar equals 50 µm. NSCLC, non-small cell lung cancer; CCK-8: cell counting kit-8; *p < 0.05, **p < 0.01, ***p < 0.001.

To determine the role of *IFI44L* gene in NSCLC, the *IFI44L* overexpression vector was constructed. SPC-A-1 and NCI-H520 were selected as the representative cells of LUAD and LUSC to study the function of *IFI44L*. Firstly, cell counting kit-8 (CCK-8) assay results showed that the growth rate of SPC-A-1 and NCI-H520 cells was significantly inhibited after overexpression of *IFI44L* (*p* < 0.05) (**Figures 10A, B**). Then, the effect of *IFI44L* overexpression on the cell migration was detected by scratch healing tests. The results showed that compared with the control group, the migration distance of cells in the *IFI44L* overexpression group was significantly reduced (*p* < 0.01) (**Figures 10C–F**). The migration and invasion of SPC-A-1 and NCI-H520 after overexpression of *IFI44L* were detected by transwell method. As shown in **Figures 10G–J**, the number of cells passing through the bio-membrane in the *IFI44L* overexpression group was significantly lower than that in the control group (*p* < 0.01). These results demonstrated that overexpression of *IFI44L* can inhibit the growth, migration, and invasion of LUSC and LUAD cells, which further proves that *IFI44L* plays a crucial role in NSCLC.

### Verification of the Prognostic Value and Immune Implication of *IFI44L* in NSCLC

**Figure 11:**
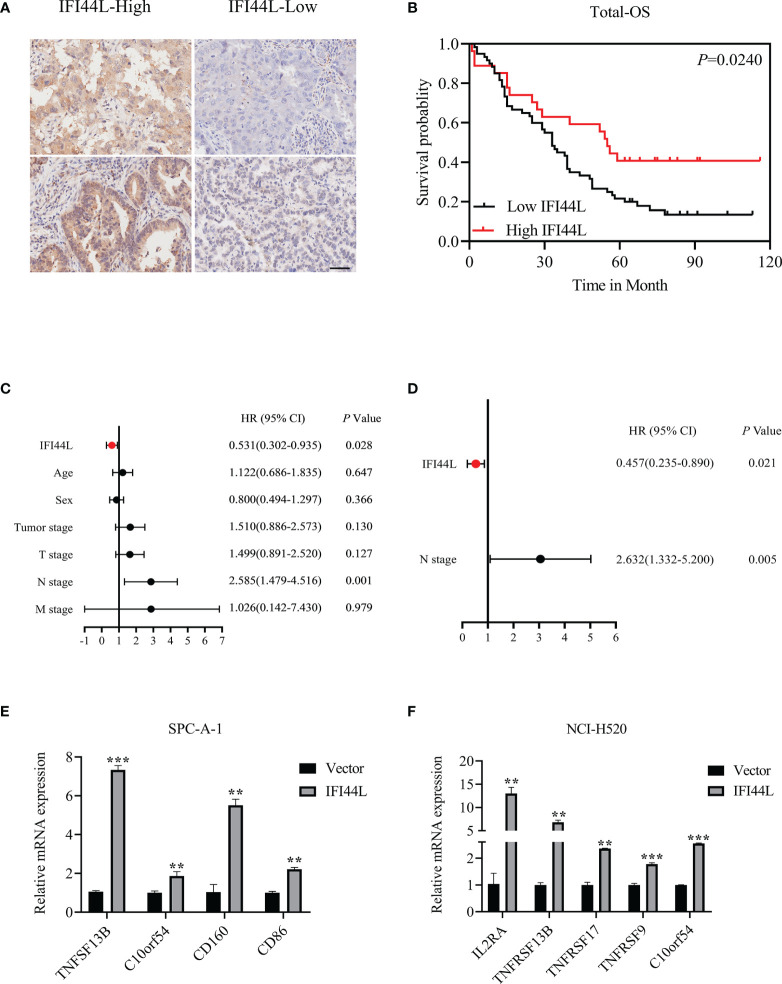
Verification of the prognostic value and immune implication of *IFI44L* in NSCLC. **(A)** Representative IHC images of IFI44L in TMA cohort with the scale bar equal to 50 µm. **(B)** The correlation of *IFI44L* expression with OS among 97 NSCLC patients. **(C)** Univariate Cox regression analysis regarding the expression of *IFI44L* and other clinical parameters. **(D)** Multivariate Cox regression analysis about the expression of *IFI44L* and clinical parameters. **(E, F)** The expression levels of the related immunomodulators were detected in NSCLC cells after overexpression of *IFI44L*. NSCLC, non-small cell lung cancer; OS, overall survival; LUAD, lung adenocarcinoma; IHC, immunohistochemistry; TMA, tissue microarray; **p < 0.01, ***p < 0.001.

In order to clarify the correlation between the expression of *IFI44L* with multiple clinical pathological parameters, a total of 97 NSCLC patients were collected to fill this gap. Representative immunohistochemical staining of IFI44L expression in NSCLC tumor tissue is shown in **Figure 11A**. Single factor analysis suggested that *IFI44L* showed an obvious negative association with AJCC stage (*χ* ^2^ = 5.853, *p* = 0.016, **Table 2**), but did not show a statistical relationship with age, gender, tumor size, T stage, N stage, and M stage. Survival analysis suggested that the high-*IFI44L* group showed better outcome than the low-*IFI44L* group (*p* = 0.024, **Figure 11B**). Next, combined with survival information, univariate Cox regression presented that *IFI44L* expression and N stage performed significant correlation with OS outcome among clinical patients (**Figure 11C**). For details, the *IFI44L* expression acts as a protective factor (HR = 0.531, 95% CI = 0.302–0.935, *p* = 0.028) and the N stage acts as a hazard factor (HR = 2.585, 95% CI = 1.479–4.516, *p* = 0.001). It is worth noting that multivariate Cox regression indicated that the *IFI44L* may act as an independent prognostic factor among lung cancer patients (HR = 0.457, 95% CI = 0.235-0.890, *p* = 0.021, **Figure 11D**).

**Table 2:** Association between clinical factors and *IFI44L* expression among NSCLC patients in the TMA cohort.

Clinical factors	Numbers	*IFI44L* expression status		*χ* ^2^	*p-*value
		High	Low		
**Total**	97	32	65		
**Age (years)**				0.004	0.947
<60	45	15	30		
≥60	52	17	35		
**Gender**				0.007	0.963
Male	54	18	36		
Female	43	14	29		
**Tumor size**				1.231	0.267
<5 cm	72	26	46		
>5 cm	25	6	19		
**T stage**				0.081	0.775
T1+T2	70	23	47		
T3+T4	25	9	16		
Unknown	2	0	2		
**N stage**				3.528	0.060
N0	43	20	23		
N1–N3	38	10	28		
Unknown	16	2	14		
**M stage**				<0.001	1.000
M0	96	32	64		
M1	1	0	1		
**AJCC Stage**				5.853	0.016^*^
Stage I–II	51	22	29		
Stage III–IV	45	9	36		
Unknown	1	1	0		

NSCLC, non-small cell lung cancer; TMA, tissue microarray; ^*^p < 0.05.

In order to verify the association between *IFI44L* and immunomodulators, we conducted an RT-qPCR experiment in the *IFI44L* overexpression cell model. After overexpression of *IFI44L*, the expression levels of these immunomodulators such as TNFSF13B and C10orf54 (associated with CD8^+^ T cells), CD160 (related to activated T cells), and CD86 (associated with regulatory T cells) increased significantly in the SPC-A-1 cell line (*p* < 0.05, **Figure 11E**). Similarly, in NCI-H520, the expression level of IL2RA (related to activated T cells and regulatory T cells), TNFSF13B, TNFRSF17 (associated with B cells), TNFRSF9 (marker of CD8^+^ T cells), and C10orf54 increased significantly (*p* < 0.05, **Figure 11F**). These results suggest that *IFI44L* can predict a better response of immunotherapy in NSCLC patients.

## Discussion

LC is an increasingly common disease that threatens public health all over the world. Diagnosis at the late stage and the limitations of traditional therapies lead to a disappointing outcome. Emerging lines of evidence indicated that immunotherapy is becoming a powerful means for LC patients (25–27). Hence, the selection and identification of novel and promising immune-related biomarkers for LC are urgently needed for clinical practice. In this study, we comprehensively analyzed the immune implication of *IFI44L* in TCGA-NSCLC samples and successfully constructed the *IFI44L*-related immunomodulator signatures to clarify its prognostic value. We found that *IFI44L* presented significant correlation with multiple TIICs, including B cells, CD8^+^ T cells, CD4^+^ T cells, macrophages, neutrophils, and dendritic cells. Interestingly, the immune infiltration levels decreased with arm-level deletion of *IFI44L*. Furthermore, a 10-immunomodulator signature was constructed in the TCGA-LUAD cohort and a 17-immunomodulator signature was established in the TCGA-LUSC cohort. Obviously, the *IFI44L*-related immunomodulator signatures could separate patients well and served as independent prognostic factors in NSCLC patients. Finally, the nomogram weighted by risk score and clinical features presented better specificity and sensitivity than a single variable in predicting OS outcome among LUAD patients.

Previous studies had suggested that *IFI44L* participates in tumor progression in certain cancers, including osteosarcoma and hepatocellular carcinoma (16, 17). As we all know, *IFI44L* played a crucial role in anti-virus processes and is competent to be a biomarker in diagnosis of viral infection (13, 28, 29). However, up to now, the functions and immune implications of *IFI44L* in NSCLC patients had not been elucidated. Therefore, we systematically analyzed the association between *IFI44L* expression and infiltration levels of multiple TIICs through bioinformatics method. The CIBERSORT results revealed that the proportions of 22 TIICs were dramatically different between the high-*IFI44L* group and the low-*IFI44L* group, whether in the TCGA-LUAD cohort or the TCGA-LUSC cohort. Consistent with the CIBERSORT results, the TIMER database showed a remarkable relationship between *IFI44L* and six TIICs, including B cells, CD8^+^ T cells, CD4^+^ T cells, macrophages, neutrophils, and dendritic cells. Intriguingly, the ssGSEA results confirmed this finding once again. *IFI44L* showed significant association with aDC, DC, iDC, macrophages, T cells, T helper cells, Tcm, Tem, Tgd, Th1 cells, Th17 cells, and TReg in TCGA-LUAD samples. Moreover, in TCGA-LUSC samples, IFI44L presented obvious correlation with aDC, B cells, CD8 T cells, cytotoxic cells, DC, eosinophils, macrophages, neutrophils, NK CD56dim cells, NK cells, pDC, T cells, T helper cells, Tcm, Tem, TFH, Th1 cells, Th2 cells, and TReg. These results indicated that *IFI44L* may be relevant to these immune cells in TME, which had been reported to be associated with NSCLC outcomes (30). Notably, the CD8^+^ T cell could be seen as an indicator to immunotherapy (31). Besides, B cells and dendritic cells were also associated with favorable survival status across human cancers (32, 33). In contrast, higher infiltration levels of Treg and macrophages M0 may induce a worse prognosis among LUAD patients (32, 34). Therefore, it is reasonable to infer that *IFI44L* played a vital role in TME and may participate in the initial stage and during the progression of NSCLC.

Due to the obvious relationship between *IFI44L* and various TIICs, we further explored the relevance between *IFI44L* and immunomodulators. A total of 44/70 immunomodulators in the TCGA-LUAD cohort and 57/70 immunomodulators in the TCGA-LUSC cohort presented significant correlation with the transcript level of *IFI44L*. As reported, the immunomodulators have tremendous and abundant functions in the TME, as well as tumor development and progression (35, 36). Among these immunomodulators, there are considerable proportions of tumor necrosis factor (TNF) and its receptor (TNFR); as the term suggest, they belong to the tumor necrosis factor super family (TNFSF) and tumor necrosis factor receptor super family (TNFRSF). As reported, the TNF is a cytokine and played an important role in inflammatory reaction, immune response, and cancer-related pathways (37, 38). In addition, TNF could induce the activity of the NF-κB pathway, thus leading to conspicuous influence in cell death and growth (39).Therefore, for the sake of comprehensively understanding the functions of *IFI44L*, future studies should not only pay attention to the role of *IFI44L* in anti-virus, stimulating the interferon and modulating the biological behavior of tumor cells, but also focus on its interaction with TME, especially its functions on the activation and motility of the TIICs, because these immune-related components act as critical determinants during tumor progression (40).

In this study, the functional enrichment analysis of *IFI44L* was performed using the GSEA method, whether in the TCGA-LUAD cohort or in the TCGA-LUSC cohort. As we expected, these results strongly suggest that *IFI44L* participates in multiple immune-related pathways, including T-cell receptor signaling pathway and B-cell receptor signaling pathway. Besides, several cancer-related pathways were also enriched, for example, the JAK/STAT signaling pathway, P53 signaling pathway, and apoptosis. As reported, JAK/STAT1, JAK/STAT3, and JAK/STAT5 are three classic pathways that play a crucial role in tumor progression. The overexpression and gene mutation of JAK family members are associated with the occurrence and development of LC patients. Xu et al. (41) found that JAK2 gene expression was upregulated in tumor tissues and presented significant association with lymph node metastasis. Upregulation of JAK2 expression could enhance the proliferation, metastasis, and invasion characteristics of tumor cells, while downregulation of JAK2 expression has the opposite effect. A further study also found JAK2 gene mutation in LUAD, suggesting that JAK2 mutation is also related to LC progression, as well as to poor prognosis and drug resistance. Another study (42) showed that JAK2 and JAK3 mutations in LC were related to the expression of programmed cell death ligand-1 (PD-L1), and patients with JAK3 gene mutations might benefit from immunotherapy. In addition, the phosphorylation level of JAK1 was significantly increased in patients with NSCLC and its high expression was associated with poor prognosis, suggesting that phosphorylated JAK1 could be used as a predictor of NSCLC treatment (43). Furthermore, another classic pathway named the NF-κB signaling pathway was also enriched among chosen immunomodulators in the TCGA-LUAD cohort. According to recent research, the NF-κB signaling pathway is involved in numerous biological processes during tumor progression, including inflammation, proliferation, apoptosis, angiogenesis, epithelial–mesenchymal transition (EMT), cancer stemness, cellular metabolism, and treatment resistance (44). It is well documented that the activated NF-κB signaling pathway could induce chronic airway inflammation, which further upregulates the functional Tregs and leads to tumor progression (45). Another research reported that the PD-L1 expression was regulated by NF-κB during EMT (46). Together, *IFI44L* may be involved in multiple cancer/immune-related pathways and play a pivotal role in TME.

Recently, emerging studies have shown that gene signatures played an important role in the prediction of cancer prognosis (47, 48). A 14 m5C-related lncRNAs signature could separate LUAD patients into a high-risk group and a low-risk group well, in relation to various TIICs (49). In addition, Zhang et al. (50) retrieved 259 ferroptosis-related genes from the FerrDb database and explored a 15-gene signature through LASSO regression in LUAD patients, with a 5-year AUC value of 0.681. Notably, another 5-gene signature in LUAD was identified and four genes were selected as predictive indicators, namely, CCNB1, CCNB2, CDK1, and AURKA (51). By combining risk score with T, N, and M, the AUC value reached 0.750 at 1 year and 0.809 at 3 years in this study. In this study, we constructed *IFI44L*-related immunomodulator signatures in LUAD and LUSC samples, respectively. Moreover, the TCGA-LUAD signature was validated in GSE72094 and a nomogram was established based on this risk signature and clinical parameters. These two signatures could both act as independent prognostic factors in NSCLC patients, whether in TCGA samples or in GEO samples. The nomogram showed good predictive ability with a C-index of 0.775 and the 1-year, 3-year, and 5-year AUC values reached 0.782, 0.825, and 0.792, respectively. Our findings suggested that the *IFI44L*-related immunomodulator signature could provide a convenient way to assess the prognosis of patients with NSCLC, which further illustrates that *IFI44L* may play a vital role in TME.

There are several limitations in this research. Firstly, our study was mainly conducted by bioinformatics methods, and most of the data applied in this work were retrieved from a public database. Although the findings were validated in another external cohort and cell trial, the clinical prospective study with a larger sample size is urgently needed to evaluate our results. Secondly, we comprehensively explored the immune implications of *IFI44L* in NSCLC; however, the mechanism between *IFI44L* and immune response is still unclear. Detailed experimental studies should focus on cell and animal research to investigate the underlying mechanism of *IFI44L* in NSCLC samples. Thirdly, although this study analyzed the association between *IFI44L* and multiple TIICs using bioinformatics methods and preliminarily explored the changes of immunomodulators in *IFI44L*-overexpressing cells by an RT-qPCR experiment, a certain number of clinical specimens and co-cultivation experiments were needed to verify these results in the future.

In summary, *IFI44L* may interact with the TME components and participate in various cancer and immune-related pathways and thus play a crucial role during NSCLC progression. A nomogram established through an *IFI44L*-related immunomodulator signature and clinical features displayed good performance in predicting prognosis outcome. It is hoped that the investigation results could supply referential information for clinical application and medical decision. Future large-scale prospective studies are needed to verify this.

## Data Availability Statement

The original contributions presented in the study are included in the article/**Supplementary Material**. Further inquiries can be directed to the corresponding author.

## Ethics Statement

The studies involving human participants were reviewed and approved by the Ethics Committee of Shanghai Outdo Biochip Company Ltd. The patients/participants provided their written informed consent to participate in this study.

## Author Contributions

WL launched the conception of this work, and conceived and wrote the manuscript. YZ and ZZ contributed equally on data processing and analysis, performed the experiments, conceived and wrote the manuscript. HC contributed to providing technical guidance during this research. JF contributed to the examination of statistical methods applied in this research. WY, JL, and SZ mainly participated in manuscript preparation and writing. All authors contributed to proofreading the manuscript and approved the final version.

## Funding

This work was supported by the National Natural Science Foundation of China (Grant No. 81872659) and the Natural Science Foundation Project of Chongqing CSTC of China (No. cstc2018jcyjAX0233).

## Conflict of Interest

The authors declare that the research was conducted in the absence of any commercial or financial relationships that could be construed as a potential conflict of interest.

## Publisher’s Note

All claims expressed in this article are solely those of the authors and do not necessarily represent those of their affiliated organizations, or those of the publisher, the editors and the reviewers. Any product that may be evaluated in this article, or claim that may be made by its manufacturer, is not guaranteed or endorsed by the publisher.

## Supplementary Material

Supplementary Figure 1 Evaluation of the proportions of 22 types of TIICs by CIBERSORT method in TCGA-NSCLC samples. **(A, B)** Violin plots indicated the differences in the TIICs distribution between high-*IFI44L* (blue) and low-*IFI44L* (red) samples in TCGA-LUAD **(A)** and TCGA-LUSC **(B)** cohort. TIICs, tumor-infiltrating immune cells; CIBERSORT, Cell Type Identification By Estimating Relative Subsets Of RNA Transcripts; TCGA, The Cancer Genome Atlas; NSCLC, non-small cell lung carcinoma; LUAD, lung adenocarcinoma; LUSC, lung squamous cell carcinoma.

Supplementary Figure 2 Evaluation of the proportions of 22 type of TIICs infiltration by CIBERSORT method between adjacent normal samples and the tumor samples. **(A, B)** Violin plots indicated the infiltration difference of TIICs between normal and LUAD samples **(A)**, as well as the LUSC samples **(B)**. The blue represents adjacent normal samples and red represents malignant samples. CIBERSORT: Cell Type Identification By Estimating Relative Subsets Of RNA Transcripts; TIICs, tumor-infiltrating immune cells; LUAD, lung adenocarcinoma; LUSC, lung squamous cell carcinoma.

Supplementary Figure 3 GSEA results showed that diverse immune/cancer-related pathways were enriched in high-*IFI44L* group. **(A, B)** *IFI44L* may be involved in multiple immune/cancer-related pathways, no matter in TCGA-LUAD samples **(A)** and TCGA-LUSC samples **(B)**. GSEA, gene set enrichment analysis; TCGA, The Cancer Genome Atlas; LUAD, lung adenocarcinoma; LUSC, lung squamous cell carcinoma.

Supplementary Figure 4 Scatter plots between *IFI44L* expression level and TIICs subsets in TCGA-LUAD samples. The Y axis represents infiltration level of immune cell subset and the X axis represents transcript level of *IFI44L*. TIICs, tumor-infiltrating immune cells; TCGA, The Cancer Genome Atlas; LUAD, lung adenocarcinoma.

Supplementary Figure 5 Scatter plots between *IFI44L* expression level and TIICs subsets in TCGA-LUSC samples. The Y axis represents infiltration level of immune cell subset and the X axis represents transcript level of *IFI44L*. TIICs, tumor-infiltrating immune cells; TCGA, The Cancer Genome Atlas; LUSC, lung squamous cell carcinoma.

Supplementary Figure 6 PPI and functional enrichment analysis of the immunomodulators collected from TCGA-LUAD samples. **(A)** PPI analysis about IFI44L-related immunomodulators through STRING online tool. **(B)** KEGG enrichment analysis of these 44 immunomodulators above mentioned. **(C)** GO enrichment analysis of these 44 immunomodulators. TCGA, The Cancer Genome Atlas; LUAD, lung adenocarcinoma; PPI, protein-protein interaction; KEGG, Kyoto Encyclopedia of Genes and Genomes; GO, Gene Ontology.

Supplementary Figure 7 Survival plot between high-risk group and low-risk group among TCGA-LUSC samples based on TCGA-LUAD signature.

## Abbreviations

LC, lung cancer; NSCLC, non-small cell lung cancer; LUAD, lung adenocarcinoma; LUSC, lung squamous cell carcinoma; ICIs, immune checkpoint inhibitors; OS, overall survival; TMA, tissue microarray; PFS, progression-free survival; TME, tumor immune microenvironment; TIICs, tumor-infiltrating immune cells; IFI44L, interferon-induced protein 44-like; ISG, interferons-stimulated gene; SLE, systemic lupus erythematosus; RSV, respiratory syncytial virus; JEV, Japanese encephalitis virus; GSEA, Gene Set Enrichment Analysis; ssGSEA, single sample Gene Set Enrichment Analysis; TCGA, The Cancer Genome Atlas; GEO, Gene Expression Omnibus; CIBERSORT, Cell type Identification By Estimating Relative Subsets Of RNA Transcripts; TIMER, Tumor IMmune Estimate Resource; PPI, protein–protein interaction; GO, Gene Ontology; KEGG, Kyoto Encyclopedia of Genes and Genomes; FDR: false discovery rate; AIC: Akaike information criterion; ROC, receiver operating characteristics; CI, confidence interval; AUC, area under curve; HR, hazard ratio; C-index, concordance index; CNV, copy number variation; NES, normalized enrichment score; aDC, activated dendritic cell; DC, dendritic cell; iDC, immature dendritic cell; Tcm, central memory T cells; Tem, effector memory T cells; Tgd, gamma delta T cells; Th1 cells, type 1 T helper cells; Th17 cells, type 17 T helper cells; TReg, regulatory T cell; NK CD56dim cells, CD56dim natural killer cell; pDC, plasmacytoid dendritic cell; TFH, T follicular helper cell; TNF, tumor necrosis factor; TNFR, tumor necrosis factor receptor; TNFSF, tumor necrosis factor super family; TNFRSF, tumor necrosis factor receptor super family; PD-L1, programmed cell death ligand-1; EMT, epithelial mesenchymal transition; CCK-8, cell counting kit-8.
