# Supplementary figures and images for: Comprehensive Analysis of Immune Implication and Prognostic Value of IFI44L in Non-Small Cell Lung Cancer
Source: Front Oncol. 2022 Jan 3;11:798425. doi: 10.3389/fonc.2021.798425 (PMC8761744; doi:10.3389/fonc.2021.798425)

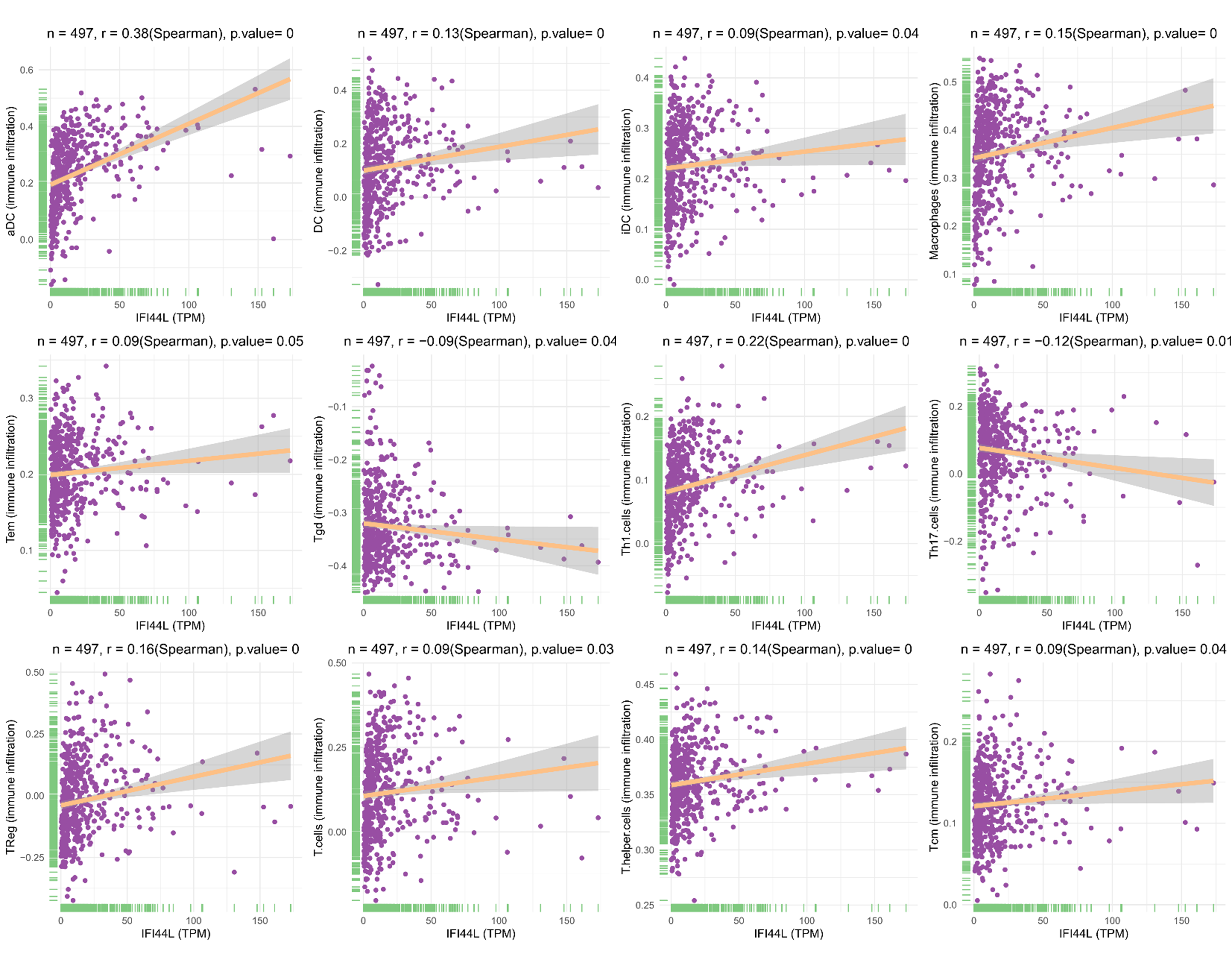

Supplement: Supplementary Figure 4 — Scatter plots between IFI44L expression level and TIICs subsets in TCGA-LUAD samples. The Y axis represents infiltration level of immune cell subset and the X axis represents transcript level of IFI44L. TIICs, tumor-infiltrating immune cells; TCGA, The Cancer Genome Atlas; LUAD, lung adenocarcinoma. [file Image_4.tif]

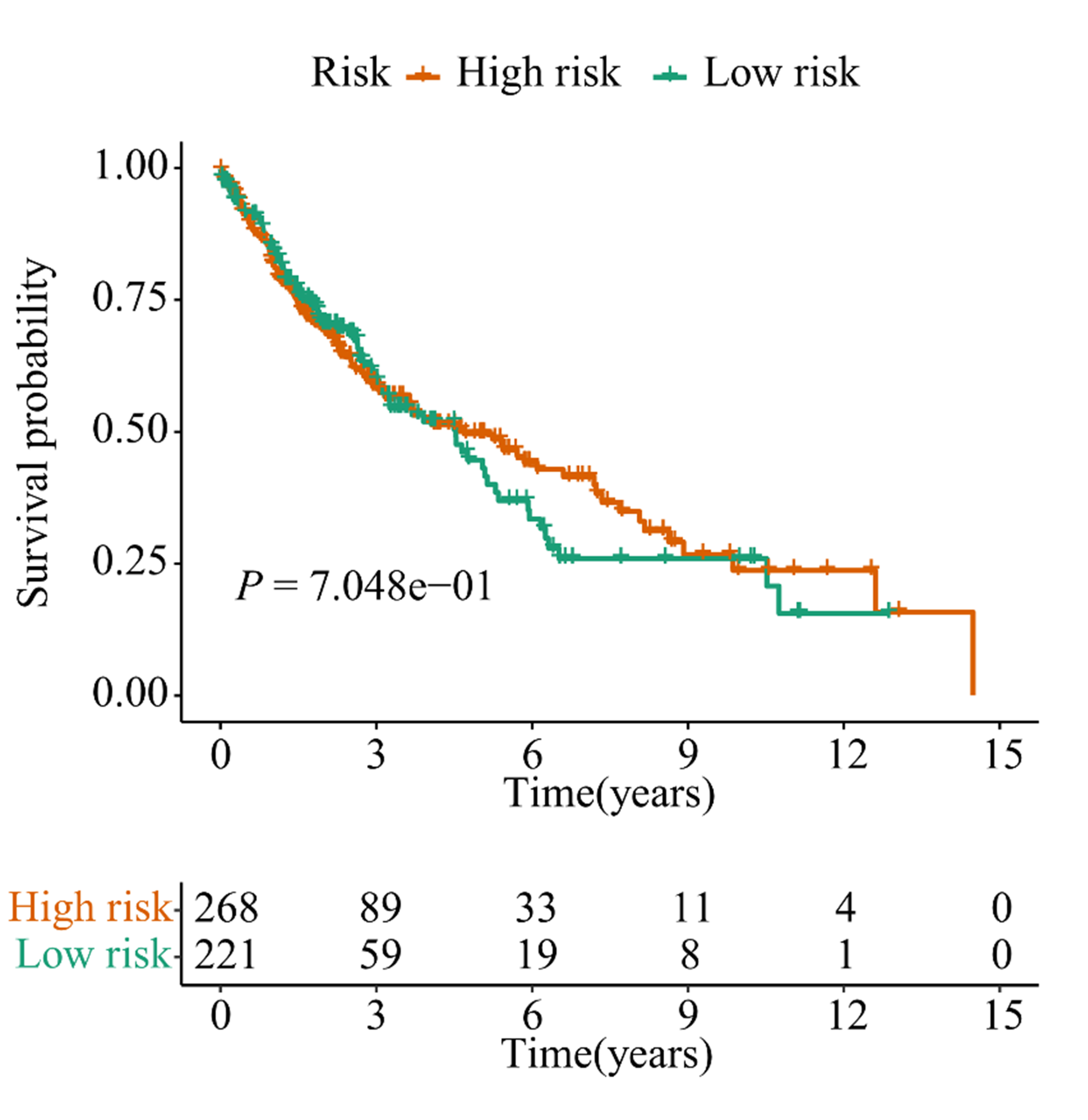

Supplement: Supplementary Figure 7 — Survival plot between high-risk group and low-risk group among TCGA-LUSC samples based on TCGA-LUAD signature. [file Image_7.tif]
