# Supplementary material for: Comprehensive Analysis of Immune Implication and Prognostic Value of IFI44L in Non-Small Cell Lung Cancer
Source: Front Oncol. 2022 Jan 3;11:798425. doi: 10.3389/fonc.2021.798425 (PMC8761744; doi:10.3389/fonc.2021.798425)
Supplement: Supplementary file 8 [file Table_1.docx]

**Supplementary Table 1** Baseline information of enrolled samples in this study.

| **Clinical characteristics** | **TCGA-LUAD cohort (n=497)** | **TCGA-LUSC cohort (n=489)** | **GSE72094 (n=398)** |
| --- | --- | --- | --- |
| Age, *x ± SD*^*^ | 65.24 ± 10.06 | 67.19 ± 8.51 | 69.36 ± 9.44 |
| Gender, *n*^**^ |  |  |  |
| Male | 228 (45.88%) | 362 (74.03%) | 176 (44.22%) |
| Female | 269 (54.12%) | 127 (25.97%) | 222 (55.78%) |
| Stage, *n* |  |  |  |
| Stage I + II | 385 (77.46%) | 395 (80.78%) | 321 (80.65%) |
| Stage III + IV | 105 (21.13%) | 90 (18.40%) | 72 (18.09%) |
| T, *n* |  |  |  |
| T 1+2 | 433 (87.12%) | 397 (81.19%) |  |
| T 3+4 | 61 (12.27%) | 92 (18.81%) |  |
| N, *n* |  |  |  |
| N0 | 321 (64.59%) | 313 (64.01%) |  |
| N1-3 | 165 (33.20%) | 171 (34.97%) |  |
| M, *n* |  |  |  |
| M0 | 331 (66.60%) | 402 (82.21%) |  |
| M1 | 24 (4.83%) | 7 (1.43%) |  |
| Smoking status, *n* |  |  |  |
| Ever |  |  | 300 (75.38%) |
| Never |  |  | 31 (7.79%) |
| Mean survival time, *x ± SD* | 2.50 ± 2.46 | 2.67 ± 2.64 | 2.17 ± 1.10 |
| Survival status, *n* |  |  |  |
| Alive | 317 (63.78%) | 279 (57.06%) | 285 (71.61%) |
| Dead | 180 (36.22%) | 210 (42.94%) | 113 (28.39%) |

^*^Continuous variables were represented by means and standard deviations (*x ± SD*); ^**^Categorical variables were represented by quantity values (*n*); TCGA: The Cancer Genome Atlas; LUAD: lung adenocarcinoma; LUSC: lung squamous cell carcinoma.
