# Supplementary material for: Comprehensive Analysis of Immune Implication and Prognostic Value of IFI44L in Non-Small Cell Lung Cancer
Source: Front Oncol. 2022 Jan 3;11:798425. doi: 10.3389/fonc.2021.798425 (PMC8761744; doi:10.3389/fonc.2021.798425)
Supplement: Supplementary file 9 [file Table_2.docx]

**Supplementary Table 2** Primer nucleotide sequence of this study.

| **Gene** | **Primer nucleotide sequence** |
| --- | --- |
| β-actin | Forward: 5’-CCACGAAACTACCTTCAACTCC-3’ |
|  | Reverse: 5’-GTGATCTCCTTCTGCATCCTGT-3’ |
| TNFSF13B | Forward: 5’-GGTCCAGAAGAAACAGGATCTTA-3’ |
|  | Reverse: 5’-CATCCCCAAAGACATGGACCT-3’ |
| C10orf54 | Forward: 5’-TCTCCATCACCATGCGCAAC-3’ |
|  | Reverse: 5’-GCAGCCGTGATGTTTTCACTA-3’ |
| CD160 | Forward: 5’-TCCATTCTATTCACAGAGACAGGG-3’ |
|  | Reverse: 5’-AAGTTTCTTTTGGCACAAGGC-3’ |
| CD86 | Forward: 5’-CCTTCCTGCTCTCTGCTAACTT-3’ |
|  | Reverse: 5’-ACAAGCTGATGGAAACGTCG-3’ |
| IL2RA | Forward: 5’-GTGGTGGGGCAGATGGTTTA-3’ |
|  | Reverse: 5’-TTGTGACGAGGCAGGAAGTC-3’ |
| TNFRSF17 | Forward: 5’-CCTCGAGTACACGGTGGAAG-3’ |
|  | Reverse: 5’-GTGACAAGAATGGTTGCGCC-3’ |
| TNFRSF9 | Forward: 5’-TTCCTCACGCTCCGTTTCTC-3’ |
|  | Reverse: 5’-AATCGGCAGCTACAGCCATC-3’ |
