# Supplementary material for: Comprehensive Analysis of Immune Implication and Prognostic Value of IFI44L in Non-Small Cell Lung Cancer
Source: Front Oncol. 2022 Jan 3;11:798425. doi: 10.3389/fonc.2021.798425 (PMC8761744; doi:10.3389/fonc.2021.798425)
Supplement: Supplementary file 10 [file Table_3.docx]

**Supplementary Table 3** Functions and coefficients of the 10 immunomodulators in TCGA-LUAD risk signature.

| **Gene symbol** | **Full name** | **Function** | **Risk coefficient** |
| --- | --- | --- | --- |
| C10orf54 | chromosome 10 open reading frame 54 | V-Type Immunoglobulin Domain-Containing Suppressor Of T-Cell Activation | 0.275866 |
| CD160 | CD160 molecule | Associated with peripheral blood NK cells and CD8 T lymphocytes with cytolytic effector activity | -0.28765 |
| CD40LG | CD40 ligand | Regulates B cell function by engaging CD40 on the B cell surface | -0.48518 |
| CD86 | CD86 molecule | Expressed by antigen-presenting cells, and it is the ligand for two proteins at the cell surface of T cells, CD28 antigen and cytotoxic T-lymphocyte-associated protein 4 | -0.43633 |
| CTLA4 | cytotoxic  T-lymphocyte-associated protein 4 | A member of the immunoglobulin superfamily and encodes a protein which transmits an inhibitory signal to T cells | -0.2302 |
| IL10 | interleukin 10 | Pleiotropic effects in immunoregulation and inflammation | -0.21303 |
| NT5E | 5'-nucleotidase, ecto (CD73) | A plasma membrane protein that catalyzes the conversion of extracellular nucleotides to membrane-permeable nucleosides | 0.193922 |
| TIGIT | T cell immunoreceptor with Ig and ITIM domains | Bind PVR with high affinity and assist interactions between TFH and dendritic cells to regulate T cell dependent B cell responses | 0.31589 |
| TNFSF13B | tumor necrosis factor (ligand) superfamily, member 13b | Play an important role in the proliferation and differentiation of B cells | 0.553219 |
| TMEM173 | transmembrane protein 173 | A major regulator of the innate immune response to viral and bacterial infections | -0.11194 |

TCGA: The Cancer Genome Atlas; LUAD: lung adenocarcinoma
