# Supplementary material for: Comprehensive Analysis of Immune Implication and Prognostic Value of IFI44L in Non-Small Cell Lung Cancer
Source: Front Oncol. 2022 Jan 3;11:798425. doi: 10.3389/fonc.2021.798425 (PMC8761744; doi:10.3389/fonc.2021.798425)
Supplement: Supplementary file 11 [file Table_4.docx]

**Supplementary Table 4** Functions and coefficients of the 17 immunomodulators in TCGA-LUSC risk signature.

| **Gene symbol** | **Full name** | **Function** | **Risk coefficient** |
| --- | --- | --- | --- |
| BTLA | B and T lymphocyte associated | A receptor that relays inhibitory signals to suppress the immune response | -0.5712 |
| C10orf54 | chromosome 10 open reading frame 54 | V-Type Immunoglobulin Domain-Containing Suppressor Of T-Cell Activation | 0.353429 |
| CD27 | CD27 molecule | Plays a key role in regulating B-cell activation and immunoglobulin synthesis | -0.60192 |
| CD48 | CD48 molecule | participates in activation and differentiation pathways in lymphocytes and other immune cells, dendritic cells and endothelial cells | -0.36787 |
| IL2RA | interleukin 2 receptor, alpha | Soluble IL2RA has been isolated and determined to result from extracellular proteolyisis. | 0.306909 |
| KLRC1 | killer cell lectin-like receptor subfamily C, member 1 | Implicated in the recognition of the MHC class I HLA-E molecules in NK cells | -0.46155 |
| LTA | lymphotoxin alpha | Mediates a large variety of inflammatory, immunostimulatory, and antiviral responses | 0.420914 |
| TMEM173 | transmembrane protein 173 | A major regulator of the innate immune response to viral and bacterial infections | -0.24701 |
| TNFRSF13B | tumor necrosis factor receptor superfamily, member 13B | Plays a crucial role in humoral immunity by interacting with a TNF ligand | 0.40972 |
| TNFRSF17 | tumor necrosis factor receptor superfamily, member 17 | May be important for B cell development and autoimmune response | 0.507039 |
| TNFRSF4 | tumor necrosis factor receptor superfamily, member 4 | Activate NF-kappaB through its interaction with adaptor proteins TRAF2 and TRAF5 | -0.38792 |
| TNFRSF8 | tumor necrosis factor receptor superfamily, member 8 | A positive regulator of apoptosis, and also has been shown to limit the proliferative potential of autoreactive CD8 effector T cells and protect the body against autoimmunity | 0.090941 |
| TNFRSF9 | tumor necrosis factor receptor superfamily, member 9 | Induce proliferation in peripheral monocytes, enhance T cell apoptosis induced by TCR/CD3 triggered activation | 0.198508 |
| TNFSF13 | tumor necrosis factor (ligand) superfamily, member 13 | A ligand for TNFRSF17/BCMA and was found to be important for B cell development | 0.323344 |
| TNFSF13B | tumor necrosis factor (ligand) superfamily, member 13b | Play an important role in the proliferation and differentiation of B cells | 0.217375 |
| TNFSF4 | tumor necrosis factor (ligand) superfamily, member 4 | Functions in T cell antigen-presenting cell (APC) interactions and mediates adhesion of activated T cells to endothelial cells | -0.22511 |
| TNFSF9 | tumor necrosis factor (ligand) superfamily, member 9 | Involved in the antigen presentation process and in the generation of cytotoxic T cells | 0.141626 |

TCGA: The Cancer Genome Atlas; LUSC: lung squamous cell carcinoma
